# Changes in Children’s Well-Being and Mental Health Across the Early School Years: Links With Academic and Social Competence

**DOI:** 10.1037/dev0001962

**Published:** 2025-04-10

**Authors:** Rory T. Devine, Louise Gray, Miryam Edwards, Mikeda Jess, Caoimhe Dempsey, Jean Heng, Mishika Mehrotra, Hana D’Souza, Elian Fink, Claire Hughes

**Affiliations:** 1 School of Psychology, Center for Developmental Science, University of Birmingham; 2 Center for Family Research, University of Cambridge; 3 School of Psychology, Cardiff University; 4 School of Psychology, University of Sussex

**Keywords:** well-being, mental health, academic attainment, social competence, longitudinal

## Abstract

The aim of the present study was to examine the relation between children’s well-being and mental health in the early years of primary school and the developmental association between well-being and mental health and children’s early social and academic skills. Two hundred fifty-two children (131 girls, *M*_age_ = 5.40 years, 80% White) and their caregivers (89.8% mothers) from the United Kingdom participated in a 1-year longitudinal study. Children completed measures of well-being, cognitive, and academic skills. Caregivers provided ratings of children’s well-being and mental health. Teachers and caregivers rated children’s social competence. Measurement models showed that well-being and mental health were distinct constructs at both time points. There were moderate levels of rank-order stability in well-being but declines in average levels of well-being with a corresponding increase in mental health difficulties. Well-being and mental health exhibited differential associations with social competence and academic performance. Initial levels of mental health predicted later academic and social competence, while gains in well-being were associated with academic skills and social competence.

## Public Significance Statement

Drawing on children’s and caregivers’ perspectives on children’s happiness at school, this study tracked 252 5-year-old children and their caregivers over 1 year and demonstrated that happiness is distinct from the absence of mental health difficulties. Children’s and their caregivers’ ratings of children’s happiness became more aligned over time, and these ratings revealed that average levels of happiness declined in the early stages of primary school. Mental health difficulties at the start of the study predicted poorer academic and social outcomes 1 year later, while gains in happiness over time were associated with better academic and social outcomes.

Children vary in how well they navigate the social and academic demands of primary school, and these differences between children matter long-term. Poor performance on measures of mathematics and reading at the start of school predicts later educational attainment (Duncan et al., 2007), and difficulties building and maintaining peer relationships in primary school predict later adult loneliness, as well as physical and mental health (Cundiff & Matthews, 2018; Matthews et al., 2019). Emerging evidence suggests that children’s well-being (i.e., their happiness and satisfaction; Ryan & Deci, 2001) and mental health are linked with both academic and social competence (Deighton et al., 2018; Yu et al., 2023). An understanding of children’s well-being is important because cross-national studies indicate that approximately one in 15 (7.6%) 5- to 9-year-old children have a mental health condition (World Health Organisation, 2022) and between 1% and 12% of 8- to 12-year-old children report low levels of satisfaction and happiness (Rees et al., 2020). Remarkably, few studies have investigated either the overlap between mental health and well-being in the early years of primary school or the developmental association between well-being and mental health and children’s early social and academic skills. The overarching aim of the present study was to address these gaps in understanding by examining both the link between well-being and mental health in the early years of primary school and the developmental association of well-being and mental health with children’s early social and academic skills.

## Are Child Well-Being and Mental Health Distinct Constructs?

According to the “traditional model,” well-being and poor mental health are two ends of the same continuum (Greenspoon & Saklofske, 2001). In other words, the absence of symptoms of mental health conditions is viewed as an indicator of well-being. By contrast, the “dual-factor model” proposes that subjective well-being and mental health are separable and make distinct contributions to children’s developmental outcomes (Greenspoon & Saklofske, 2001; Petersen et al., 2020; S. M. Suldo & Shaffer, 2008). In line with this view, the World Health Organisation (2022) described mental health as comprising two dimensions representing (a) well-being (i.e., the degree of positive affect and satisfaction) and (b) symptoms of mental health conditions. So, while individuals experiencing symptoms of mental health conditions are more likely to report lower levels of well-being, it is possible to experience high levels of well-being in the context of mental health conditions (World Health Organisation, 2022).

Several studies have investigated the links between mental health and well-being in children and adolescents. For example, data from the U.K. Millennium Cohort Study revealed modest negative correlations between caregiver-reported psychopathology and children’s self-reported happiness, both at age 11 years (Patalay & Fitzsimons, 2016) and at age 14 years (Patalay & Fitzsimons, 2018). Results from age 14 years showed that the correlation between subjective well-being and psychopathology was stronger when both constructs were rated by the same informant, but still not entirely overlapping (Patalay & Fitzsimons, 2018). Data from the Avon Longitudinal Study of Parents and Children in the United Kingdom revealed similarly modest associations between caregiver-rated externalizing and internalizing and self-rated school enjoyment (an indicator of well-being) at ages 10–11 years and 13–14 years (Cadman et al., 2021). The weak-to-moderate correlations between mental health and well-being suggest these two dimensions are distinct, supporting the dual-factor model in middle childhood and early adolescence. However, existing work on the correlations between mental health and well-being has relied on using observed summed scores, which reflect both the underlying true score and measurement error, such that correlations between constructs may be underestimated (Brown, 2015). Latent variable modeling provides a method for testing the overlap or distinctiveness of error-free true scores and comparing the fit of competing measurement models to a given data set (i.e., a “traditional” one-factor model vs. a dual-factor model).

In children, mental health is typically measured using indicators of internalizing (e.g., depressive symptoms, anxiety symptoms, emotional difficulties) and externalizing problems (e.g., conduct problems, impulsivity, oppositional behavior) with standardized scales completed by caregivers (e.g., parents, teachers; Stone et al., 2010). Reflecting the long-standing emphasis on a deficit approach to mental health, there is less consensus on how best to measure well-being in children (S. Suldo et al., 2011). Moreover, existing studies of children’s well-being have largely focused on middle childhood and adolescence, using self-reported or informant-rated indicators of happiness (e.g., life satisfaction, positive affect) that focus on either global or specific domains (e.g., school; Morris et al., 2021; Patalay & Fitzsimons, 2018; Rees et al., 2020; S. Suldo et al., 2011). As a result, the links between well-being and mental health in younger children are poorly understood.

A key barrier to extending the developmental scope of research on well-being and mental health was the lack of age-appropriate measures of well-being for younger children. To address this challenge, Ford et al. (2012) developed a child-friendly seven-item self-report measure of well-being called “How I Feel About My School” (HIFAMS). The HIFAMS has shown acceptable levels of test–retest and internal consistency reliability, as well as modest cross-sectional correlations between observed summed scores for subjective well-being and caregiver- and teacher-rated mental health, echoing findings from older children (Allen et al., 2018). The first aim of our study was to examine the relations between mental health and well-being in early primary education by using latent variable analysis to compare the fit of competing models (i.e., one- vs. two-factor models) and the role of informant effects (i.e., caregiver and child ratings).

## Stability and Continuity in Child Well-Being and Mental Health in the Early School Years

Numerous studies have investigated stability (i.e., rank-order consistency) and continuity (i.e., group mean consistency) in mental health across childhood (Bornstein et al., 2017; Patalay & Fitzsimons, 2018), but comparatively little is known about stability and continuity in young children’s subjective well-being. In early adolescence, longitudinal data suggest that individual differences in subjective well-being exhibit weaker rank-order stability than measures of psychopathology, suggesting that ratings of subjective well-being capture a stable sense of happiness but may be more influenced by transient feelings than mental health condition symptoms (Cadman et al., 2021; Patalay & Fitzsimons, 2018). Patterns of stability in well-being observed in adolescence cannot be extrapolated to earlier developmental periods. Young children show a propensity to live in the moment, both because autobiographical memory emerges slowly across the life span (Fivush, 2011) and because children’s limited working memory capacity constrains their ability to engage in episodic future thinking (Ferretti et al., 2018). On this basis, young children’s ratings of well-being may be less stable over time than adolescents’ and less stable than caregiver-rated child well-being. Challenging this view, 4-year-old children’s self-reported enjoyment at school showed moderate levels of rank-order stability over 6 months (Jirout et al., 2023).

Alongside assessing the stability of individual differences, we were also interested in tracking continuity in mean levels of children’s well-being and mental health. Large-scale cross-sectional work on subjective well-being in middle childhood indicates that child-reported well-being declines between the ages of 10 and 12 years (Rees et al., 2020). Mirroring these findings, longitudinal data from early adolescence indicate within-person decreases in average levels of subjective well-being between 11 and 14 years of age (Patalay & Fitzsimons, 2018). While it is not possible to generalize patterns of discontinuity from early adolescence to early childhood, environmental factors might influence average levels of subjective well-being in early childhood. The move from “Reception” to “Year 1” of primary school (i.e., from the first to the second year of formal schooling in England, equivalent to the transition from pre-Kindergarten to Kindergarten in the United States) is marked by a move from the “Early Years Foundation Stage,” which is characterized by an active play-based approach to learning, to “Key Stage 1,” which is characterized by a more structured, formal curriculum and may bring challenges that are sometimes overlooked (Fisher, 2010). However, it would be wrong to assume that these new demands necessarily lead to reduced well-being. Indeed, greater levels of autonomy could lead to gains in subjective well-being (Ryan & Deci, 2001).

There are likely to be marked individual differences in the degree of change in well-being over time. Alongside the paucity of research examining rank-order stability and mean-level continuity in subjective well-being, existing studies have yet to examine either variation in the degree of change in children’s well-being or whether *changes* in well-being are related to *changes* in children’s mental health. Our second aim was therefore to examine stability and continuity in subjective well-being and mental health across the early primary years and to investigate the extent to which *changes* in well-being relate to *changes* in children’s mental health. To this end, we used latent change score models to investigate baseline correlations between mental health and well-being, cross-domain associations between initial levels of well-being and changes in mental health, between initial levels of mental health and changes in well-being, and cross-domain coupling in changes in both constructs (Kievit et al., 2018).

## Well-Being, Mental Health, and Success at School

According to the dual-factor model, mental health and well-being make distinct contributions to children’s development (S. M. Suldo & Shaffer, 2008). Research on the correlates of mental health and subjective well-being lends support to the dual-factor model. First, longitudinal data indicate that these constructs have distinct predictors. Among 11-year-old children, cognitive ability and parental mental health each uniquely predict child mental health more strongly than they predict subjective well-being, while social relationships are more strongly related to subjective well-being than to child mental health (Patalay & Fitzsimons, 2016). Moreover, subjective well-being and mental health make unique contributions to children’s academic achievement and social competence. Specifically, cross-sectional studies of adolescents show that those with poor mental health and high subjective well-being had better social functioning than those with poor mental health and low subjective well-being (S. M. Suldo & Shaffer, 2008). Over and above potential confounds, such as early cognitive ability and parental socioeconomic status (SES), mental health (i.e., internalizing and externalizing) and well-being (i.e., enjoyment of school) made unique contributions to academic outcomes at age 16 (Cadman et al., 2021; Morris et al., 2021). Mental health and subjective well-being were as strong as SES as predictors of academic success (Morris et al., 2021). Subjective well-being and mental health may therefore each contribute uniquely to children’s social and academic “success” at school.

Extending the focus of this work to the first years of primary school is important because problems in early academic performance and social competence each cast a long shadow (Duncan et al., 2007). To isolate unique associations between subjective well-being, mental health, and social and academic outcomes, it is important to include potential confounds such as cognitive ability, parental SES, and gender (e.g., Morris et al., 2021). Our third aim was to extend existing work by examining the relations between initial levels and changes in children’s subjective well-being and mental health and early indicators of social and academic skills. We used structural equation modeling to examine the unique associations between well-being and mental health and academic performance and social competence, adjusting our estimates for potential confounds such as earlier cognitive ability, parental SES, and child sex.

## Summary of Aims

In summary, our study had three aims. The first aim was to use latent variable modeling to compare the fit of competing models of mental health and well-being (i.e., one- vs. two-factor models) and the role of informant effects (i.e., caregiver and child ratings). Our second aim was to examine stability and continuity in subjective well-being and mental health across the first year of formal education and to investigate how changes in well-being relate to changes in children’s mental health. Our third aim was to investigate the relations between initial levels and changes in children’s well-being and mental health and early indicators of social and academic skills in the first years of formal education.

## Method

### Transparency and Openness

We report how we determined our sample size, all data exclusions, all manipulations, and all measures in the study. All data and research materials are available from the U.K. Data Service (Hughes et al., 2024). The analytic code is available in the Supplemental Materials. The analyses were not preregistered. We analyzed the data using a latent variable framework in *Mplus* Version 8 (Muthèn & Muthèn, 2017).

### Participants

Children and their primary caregivers were recruited from across England in the Spring/Summer 2021 via targeted mailings to primary schools and paid targeted social media advertising. To participate in the study, children were required to be enrolled in the first year of primary school in England (“Reception”) and have no history of developmental delay. In England, children typically start the reception year of primary school in the September after their fourth birthday. The primary caregiver and participating child had to be able to communicate in English. We sought to recruit 250 children into the longitudinal study (see the Supplemental Materials for sample size justification). Just under 500 caregivers expressed an interest in learning more about the study (*N* = 494), and 260 of these families agreed to participate (52.6%). Of these 260 families, five families did not provide sufficient information to establish eligibility, one child was not attending Reception, and two families planned to leave England before follow-up. At Time 1, 252 children (131 girls) aged 5.40 years (*SD* = 0.31) and their caregivers (89.8% mothers, *M*_age_ = 38.63 years, *SD* = 4.66) participated in the study. Children were predominantly from two-parent heterosexual households (92.1%). Caregivers were highly educated (83.7% had degree-level education). On the subjective ladder of social status, 74.3% of caregivers rated themselves as 6/10 or above on a 10-point scale where 1 indicated the lowest levels of education, income, and status and 10 indicated the highest levels of education, income, and status. Following the U.K. Census ethnic group categories, 80% of children were identified as “White,” 13% as “Mixed or multiple ethnicities,” 6% as “Asian,” and 1% as “Black.”

### Procedure

Children and their primary caregivers were seen on two occasions approximately 12 months apart (mean interval = 12.36 months, *SD* = 1.08 months) using a remote assessment protocol to mitigate the spread of COVID-19. Data collection was timed to take place when children had completed at least one term in Reception year and then again after completing at least one term in Year 1. At both time points, following written consent, caregivers participated in a remote interview with a graduate researcher lasting approximately 15–20 min and then completed an online questionnaire pack. During this initial meeting, researchers checked for connectivity issues and ensured families had all necessary research materials (i.e., access to a desktop computer or device with a working keyboard, safe receipt of a package that contained supporting materials including stickers and a sticker chart). Children then participated in a remote testing session using videoconference software led by two graduate researchers with a caregiver present. The remote testing session followed a standardized protocol and included a battery of tasks to measure cognitive skills (i.e., executive function, verbal ability, theory of mind), self-concept, and subjective well-being. These sessions lasted approximately 45–60 min. Children were given rest breaks and received rewards (e.g., stickers) for completing each task regardless of task outcome. No feedback on task performance was given during the testing session. Families received a voucher (£10) as a gift for participating in each wave of the study. Teachers were invited to complete a short questionnaire about each study child. Ethical approval for the study was obtained from the Research Ethics Committees at the University of Birmingham and University of Cambridge.

### Measures

#### Children’s Well-Being

At Time 1 and Time 2, primary caregivers and children completed the HIFAMS questionnaire (Ford et al., 2012). Using a standardized script, children were asked by the interviewer to rate how happy they feel during seven different aspects of the school day (e.g., “When I am in the classroom, I feel …,” “When I am in the playground, I feel …”). Children selected from three choices (i.e., “Not happy,” “OK,” “Happy”) using three colored emoticons. The caregiver version of HIFAMS was administered via online questionnaire. Caregivers rated how the target child feels during the same seven aspects of the school day using a 5-point rating scale (i.e., “Very Unhappy,” “Unhappy,” “Neither Happy nor Unhappy,” “Happy,” “Very Happy”). Given the negatively skewed distribution of item-level responses for children (i.e., at Time 1, 59%–91% said they were “Happy” on each item) and caregivers (i.e., at Time 1, 82%–96% said they were “Happy” or “Very Happy” on each item), we recoded items into binary scores. Children’s scores were recoded as “Not Happy/OK” (0) and “Happy” (1). Caregivers’ scores were recoded as “Not Very Happy” (0; i.e., all responses that were not “Very Happy”) and “Very Happy” (1). Total scores for children and caregivers could therefore range between 0 and 7.

#### Children’s Mental Health

Primary caregivers completed the *Strengths and Difficulties Questionnaire* (SDQ; Goodman et al., 2000) at Time 1 and Time 2. The SDQ is used in research on children’s mental health and shows evidence of internal consistency and test–retest reliability as well as concurrent validity with more lengthy measures of psychopathology and clinician diagnoses (Stone et al., 2010). The SDQ consisted of 25 items divided into five subscales: emotional problems (e.g., “Often unhappy, downhearted”), conduct problems (e.g., “Often fights with other children”), hyperactivity (e.g., “Easily distracted, concentration wanders”), peer problems (e.g., “Rather solitary, tends to play alone”), and prosocial skills (e.g., “Shares readily with other children”). Each item was scored on a 3-point rating scale (i.e., “Not True,” “Somewhat True,” “Certainly True”). Summed scores for each subscale had a possible range of 0–10, with high scores on each scale indicating greater levels of difficulty (note that in the Prosocial scale, higher scores indicated fewer difficulties). See Supplemental Table S3 for reliability. Based on U.K. normative data (see https://www.sdqinfo.org), the proportion of children scoring in the “average” range was 83.6% for emotional problems, 72% for conduct problems, 78.9% for hyperactivity, and 79.7% for peer problems.

#### Academic Skills

Academic skills were measured using a multimethod, multi-informant approach. At Time 2, children completed two direct assessments to capture number skills and reading ability. In the *Numeracy Screener* (Nosworthy et al., 2013), children decided which of two numbers (ranging from 1 to 9) was greater in a series of 56 symbolic and 56 nonsymbolic pairs. The total number of correct items was summed together and age residualized. In the Sight Word Efficiency subtest of the *Test of Word Reading Efficiency* (Torgesen et al., 1999), children were given 45 s to read aloud a list of words. The total number of correct words was summed together and age residualized.

Caregivers and teachers reported on children’s academic performance. Caregivers completed the *Competence and Coping Questionnaire* (Mischel et al., 1988), in which they rated how their child was performing academically relative to their peers along a 7-point scale (e.g., “Not as strong as most peers” to “Much stronger than most peers”). Teachers rated children’s performance on six skills from the National Curriculum (i.e., word reading, comprehension, writing, number knowledge, addition/subtraction, geometry) relative to their classmates using a 7-point scale (i.e., “Not as strong academically as most peers” to “Much stronger academically than most peers”). Ratings were averaged to give a possible score ranging from 0 to 6 (see Supplemental Table S3 for reliability).

#### Social Competence

Social competence was measured using caregiver and teacher reports at Time 2. Caregivers completed the Social Skills Questionnaire from the *Social Skills Improvement System* (Gresham & Elliott, 2008). Caregivers rated 46 items capturing a range of social skills on a 4-point scale (i.e., “Never,” “Seldom,” “Often,” “Always”). Summed total possible scores ranged from 46 to 184 (see Supplemental Table S3 for reliability). In the *Competence and Coping Questionnaire* (Mischel et al., 1988), caregivers rated their child’s skill at maintaining friendships and getting along with peers along a 7-point scale (e.g., “Not very skilled” to “Very skilled”). Teachers assessed children’s peer social competence using the *Peer Social Maturity Scale* (Peterson et al., 2007) across eight items using a 7-point scale (e.g., “Very much less mature than peers” to “Very much more mature than peers”). Item scores were averaged to give a total possible score ranging from 0 to 6 (see Supplemental Table S3 for reliability).

#### Covariates

To measure individual differences in verbal ability, children completed the Receptive Vocabulary subtest of the *Wechsler Preschool and Primary Scale of Intelligence* (Rust, 2003). Children were asked to point to one picture (from a selection of four choices) to match a word read aloud by the examiner. The summed total scores were age residualized.

Children completed three tasks to measure executive function. The *Backwards Animal Span* task was designed to measure working memory. The examiner showed children short sequences of animal pictures (ranging from two animals to four animals) and said the name of each animal aloud (e.g., horse, snail). Children were asked to repeat each sequence in reverse order (e.g., snail, horse). Following two practice trials, children completed two trials of each length (i.e., 2, 3, and 4 one-syllable animals). Summed total scores had a possible range of 0 to 6. The *Head Toes Knees Shoulders* (Ponitz et al., 2009) task was developed as an assessment of inhibition and cognitive flexibility. In the first two phases, children were required to follow a single rule and inhibit a prepotent response (e.g., when the examiner says “head,” the child had to touch their toes and vice versa). There were four practice trials followed by 10 test trials in each phase scored as correct (1) or incorrect (0). In the third phase, children were required to learn two rules and override prepotent response. Specifically, children were required to touch their toes when the examiner said “shoulders” (and vice versa) and touch their knees when the examiner said “head” (and vice versa). This final phase consisted of four practice trials followed by 10 test trials. Summed total scores had a possible range of 0 to 30.

The *Fish Flanker Task* (Rueda et al., 2004) was designed to assess inhibition and was administered remotely using Gorilla Experiment Builder (Anwyl-Irvine et al., 2020). Children were required to “feed the fish” pictured in the middle of the screen ignoring fish on either side of this target fish. In the congruent (control) trials, all fish faced in the same direction. In the incongruent (inhibition) trials, children had to ignore the surrounding fish because the fish in the middle faced in the opposite direction to the other fish. Children completed six practice trials with feedback followed by 36 test trials. Each trial was preceded by a 500-ms fixation stimulus and remained on the screen until the participant responded (for a maximum of 2,500 ms). There were 17 incongruent and 19 congruent trials administered in a fixed pseudorandom order. Responses over 2,500 ms and under 250 ms were scored as “failed” trials. We calculated the rate-correct score for each condition by summing together the total number of correct trials in each condition and dividing this by the total time taken (i.e., the sum of all reaction times). This yielded a total correct trials/second for each condition.

Caregivers completed the *Ladder of Subjective Social Status* (Singh-Manoux et al., 2003) to capture differences in SES. Caregivers indicated their placement on a 10-step ladder where the top represented those with the best education, income, and employment and the bottom those with the worst.

### Analytic Strategy

We analyzed the data using a latent variable framework in *Mplus* Version 8 (Muthèn & Muthèn, 2017). We evaluated model fit using three standard criteria: a root-mean-square error of approximation of <.08, a comparative fit index of >.90, and a Tucker–Lewis index of >.90 (Brown, 2015). In nested model comparisons, we used three criteria to test changes in model fit: a significant increase in χ^2^, a decrease in comparative fit index of ≥0.01, and an increase in root-mean-square error of approximation of ≥0.015 (Luong & Flake, 2023; Roos & Bauldry, 2022).

Children were spread across 135 primary schools: 98 children were the only child participating from their school, and 154 children were recruited from 37 schools with more than one participating child (median cluster size = 3, range = 2–9 children). Across the whole sample, each of the 135 schools contributed an average of 1.87 children (*Mdn* = 1, range = 1–9). We calculated intraclass correlations for each of the main study variables by estimating intercept-only multilevel models in *Mplus* with school as a clustering variable (Finch & Bolin, 2017). Multilevel modeling was not warranted because intraclass correlations ranged from 0.005 to 0.046, indicating a negligible impact of clustering on the data (Finch & Bolin, 2017).

**Figure 1 fig1:**
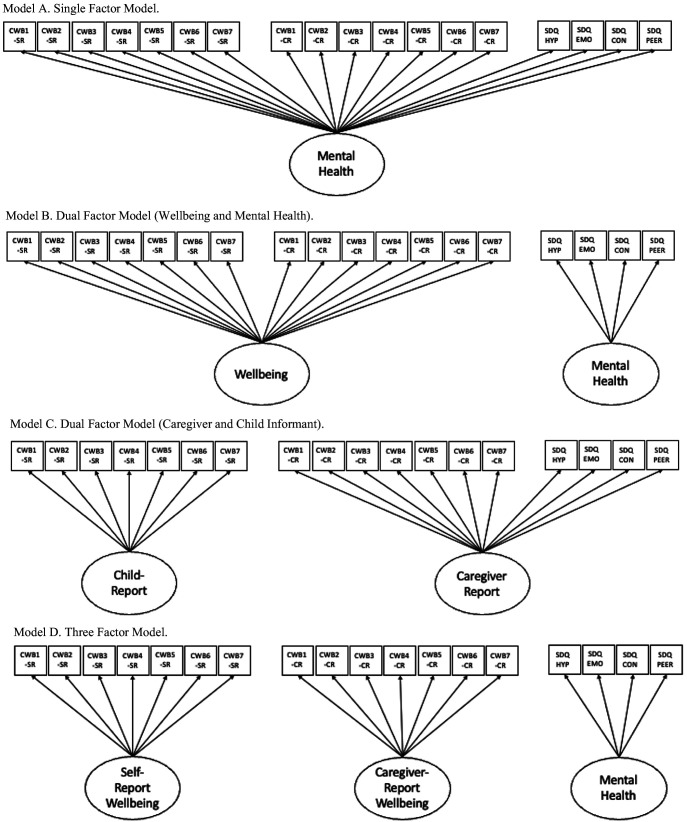
Hypothetical Measurement Models for Well-Being and Mental Health *Note*. Note that in Models B–D, we assumed that the latent factors would be correlated. CWB = child well-being; SR = self-report; CR = caregiver report; SDQ = Strengths and Difficulties Questionnaire; HYP = hyperactivity; EMO = emotional problems; CON = conduct problems; PEER = peer problems.

Our first aim was to examine whether children’s well-being and mental health were overlapping or distinct constructs. To this end, we used confirmatory factor analysis to compare the fit of competing measurement models (see Figure 1). Given that the HIFAMS used categorical responses, we adopted a mean- and variance-adjusted weighted least squares estimator (WLSMV; Roos & Bauldry, 2022). We set the metric of the latent factor using the lead indicator and freely estimated the latent factor variance. For each model, we inspected the modification indices for areas of strain. As the models were not nested, it was not possible to compare models directly. Furthermore, because the models were estimated using WLSMV, we did not obtain Akaike information criterion or Bayesian information criterion values. We therefore selected the best model using the overall fit indices and through interpretation of parameter estimates. We estimated the reliability of the latent factors using the omega coefficient (McNeish, 2018).[Fig fig1]

Our second aim was to examine rank-order stability and mean-level changes in children’s well-being and mental health. We first assessed whether the models for well-being and mental health exhibited measurement invariance over time and tested rank-order stability in each construct using autoregressive models. By testing measurement invariance, we sought to establish whether the items measuring well-being and mental health captured their respective constructs in the same way over time (Luong & Flake, 2023). We used univariate latent change score models to estimate the initial levels, means, and variances for changes in child- and caregiver-rated well-being and mental health (Kievit et al., 2018). We used bivariate latent change score models to examine longitudinal associations between child- and caregiver-rated well-being and mental health (Kievit et al., 2018).

Our third aim was to investigate the association between well-being and mental health and children’s social and academic skills in the first years of formal education. We extended our latent change score models by regressing social competence and academic abilities onto initial levels and changes in children’s well-being and mental health. We controlled for potential confounds by regressing social competence and academic abilities onto gender, SES, initial verbal ability, executive function, and prosocial behavior.

### Missing Data

Recruitment and data collection for Time 1 of the study took place in 2021 during the COVID-19 pandemic, when there were restrictions on social interactions and disruptions to education in England. Two hundred twenty-three out of 252 families participated at Time 1 and Time 2 (88.5%). Teacher questionnaires were available for 154 out of 223 children at Time 2 (69%), and caregiver questionnaires were available for 192 out of 223 children at Time 2 (86%). Table 1 shows the degree of missingness for each measure. We used logistic regression with robust maximum likelihood estimation in *Mplus* to compare the characteristics of children who took part on two occasions with those who participated at Time 1 only. To this end, we regressed a dummy variable (0 = Time 1 only, 1 = Time 1 and Time 2) onto child age, gender, caregiver SES, child verbal ability, and child well-being (see Supplemental Table S1). We also compared the characteristics of children whose teachers completed a questionnaire to those whose teachers did not complete a questionnaire (Supplemental Table S1). There were no differences between children in either analysis. Under the assumption that data were missing at random, we handled missing data by imputing 50 data sets using a Markov Chain Monte Carlo approach and a mean- and variance-weighted least squares estimator in *Mplus* (Muthèn & Muthèn, 2017; Woods et al., 2021). The imputation process included all variables that appeared in subsequent models (see the Supplemental Materials).[Table tbl1]

## Results

### Descriptive Statistics and Data Reduction

Table 1 shows the descriptive statistics for the main study variables. The Supplemental Materials describe the latent factor scores and reliability estimates for academic skills (Ω = .80), social competence (Ω = .75), and executive function (Ω = .50).

### Modeling Individual Differences in Children’s Well-Being and Mental Health

**Figure 2 fig2:**
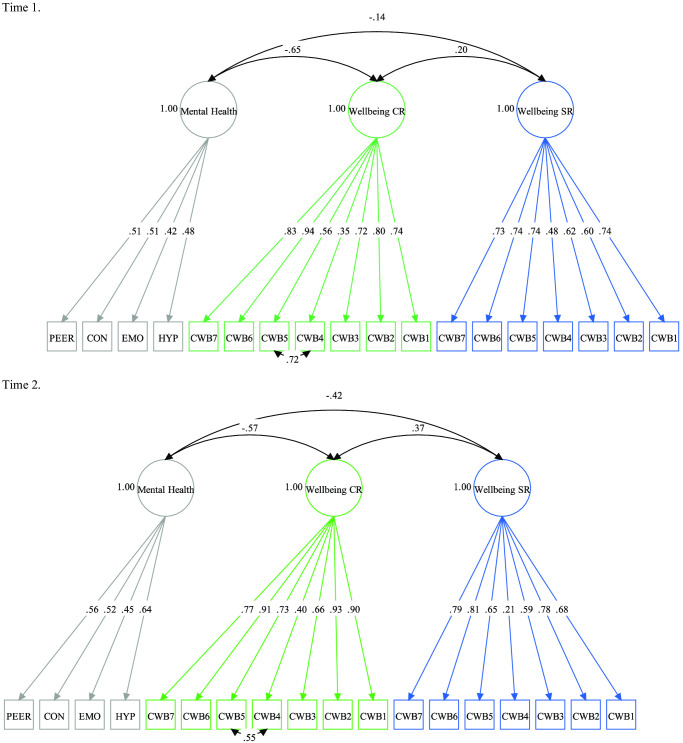
Best Fitting Measurement Model at Time 1 and Time 2 *Note*. Path diagrams show completely standardized WLSMV estimates. CWB = child well-being; CR = caregiver rated; SR = self-rated; HYP = hyperactivity; EMO = emotional problems; CON = conduct problems; PEER = peer problems; WLSMV = mean- and variance-adjusted weighted least squares estimator. See the online article for the color version of this figure.

Our first aim was to examine whether children’s well-being and mental health were overlapping or distinct constructs. Table 2 shows the model fit statistics for each model at Time 1 and Time 2. A three-factor model was the best fitting model at Time 1 and Time 2, indicating that children’s well-being and mental health were distinct but related constructs (Figure 2). Interestingly, the model in which caregiver- and child-rated well-being loaded onto one factor did not fit the data at Time 1 or Time 2, suggesting that caregiver ratings of well-being and child ratings of well-being captured related but distinct constructs. Associations between child-rated well-being (T1, Ω = .85; T2, Ω = .84) and caregiver-rated well-being (T1, Ω = .88; T2, Ω = .91) were weak at Time 1, *r* = .20, *p* = .017, and moderate at Time 2, *r* = .37, *p* < .0001. Child-rated well-being was not correlated with caregiver-rated mental health at Time 1 (Ω = .55), *r* = −.14, *p* = .186. However, at Time 2, caregiver-rated mental health (Ω = .63) showed a moderate negative association with child-rated well-being, *r* = −.42, *p* < .0001. Caregiver-rated well-being was strongly correlated with mental health at Time 1, *r* = −.65, *p* < .0001, and Time 2, *r* = −.57, *p* < .0001. Based on these analyses, we examined child-rated well-being, caregiver-rated well-being, and caregiver-rated mental health as separate constructs.[Table tbl2][Fig fig2]

### Stability and Continuity in Well-Being and Mental Health

Our second aim was to examine rank-order stability and mean-level continuity in children’s well-being and mental health across the first year of formal education. We first examined longitudinal measurement invariance in each construct (see Supplemental Table S2 for model fit indices). Having established measurement invariance for caregiver-rated child well-being and mental health and partial invariance for child-rated well-being, we examined the rank-order stability of individual differences in each construct using an autoregressive model. We regressed each latent construct at Time 2 onto the corresponding construct at Time 1 and permitted each latent factor to correlate within time points. This model, without cross-lagged paths, provided an adequate fit to the data (Table 2, Model 9). There was moderate rank-order stability in each latent factor over time. There were no significant differences between the rank-order stability of child-rated well-being, std. est. = .61, *p* < .0001, and caregiver-rated well-being, std. est. = .68, *p* < .0001, χ^2^(1) = 0.84, *p* = .36, or between the stability of child-rated well-being and caregiver-rated mental health, std. est. = 0.61, χ^2^(1) = 0.02, *p* = .88. Finally, there were no differences in rank-order stability between caregiver-rated well-being and mental health, χ^2^(1) = 0.78, *p* = .38.

**Figure 3 fig3:**
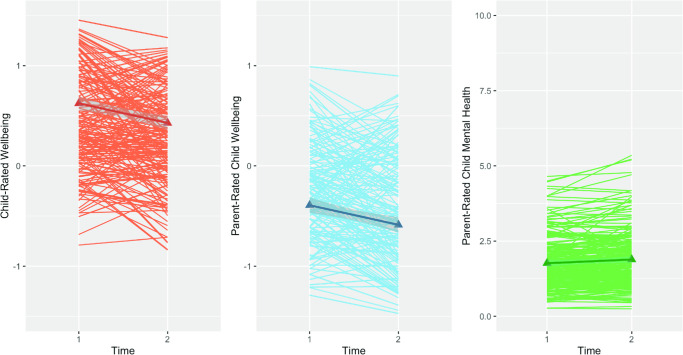
Spaghetti Plots Depicting Changes in Latent Factor Scores for Child-Rated Well-Being, Caregiver-Rated Well-Being, and Mental Health From Time 1 to Time 2 *Note*. See the online article for the color version of this figure.

Next, we examined latent mean changes in each construct. Univariate latent change score models provided a good fit to the data for child self-rated well-being, caregiver-rated child well-being, and child mental health (Table 2, Models 10–12, Figure 3). On average, there was a significant mean decrease in child-rated well-being as indicated by the mean latent change score, est. = −0.205, *SE* = 0.08, *p* < .01, as well as significant interindividual differences in the degree of change over time, est. = 0.442, *SE* = 0.10, *p* < .0001. Consistent with this pattern, mean caregiver-rated child well-being also decreased over time, as indicated by the mean latent change score, est. = −0.252, *SE* = 0.07, *p* < .0001, and again there were marked interindividual differences in change over time, est. = 0.529, *SE* = 0.11, *p* < .0001. Mean latent change scores indicated significant increases in caregiver-rated child mental health difficulties over time, est. = 0.157, *SE* = 0.08, *p* = .038, with significant interindividual differences in latent change scores, est. = 0.461, *SE* = 0.17, *p* = .007.[Fig fig3]

Next, we examined the association between initial levels and changes in child- and caregiver-rated well-being and mental health using bivariate latent change score models (Kievit et al., 2018). In the first model, we examined the longitudinal associations between child-rated well-being and caregiver-rated mental health (see Table 2, Model 13). There were moderate–strong associations between changes in well-being and changes in mental health. Gains in well-being were associated with declines in mental health problems, std. est. = −.51, *z* = −3.797, *p* < .0001. In the second bivariate model, we examined the longitudinal associations between caregiver-rated well-being and caregiver-rated mental health (see Table 2, Model 14). In contrast to the first model, there were significant associations between changes in well-being and changes in mental health, std. est. = −.19, *z* = −2.598, *p* < .01. These models suggest well-being and mental health are distinct but correlated constructs.

### Well-Being and Mental Health as Predictors of Academic Skills and Social Competence

Our third aim was to examine the association between child well-being and mental health and children’s social and academic skills at the end of the first year of school (Table 2, Models 15–20). Table 3 shows the results of models depicting relations between initial levels and changes in well-being and mental health and children’s social and academic skills, controlling for gender, SES, and Time 1 verbal ability, executive function, and prosocial behavior. Academic skills at Time 2 were significantly negatively associated with initial levels of caregiver-rated mental health but not with initial levels of caregiver- or child-rated well-being. Academic skills were not significantly associated with changes in caregiver-rated mental health or caregiver-rated child well-being. In contrast, increases in children’s subjective well-being from Time 1 to Time 2 were positively linked with academic skills at Time 2. Social competence at Time 2 was uniquely predicted by initial levels of caregiver-rated child mental health and child well-being such that mental health difficulties at Time 1 predicted poorer social competence 1 year later and positive perceptions of child well-being were associated with better social competence 1 year later. Over and above these initial levels, changes in mental health and changes in caregiver-rated child well-being were uniquely associated with children’s social competence at Time 2. Specifically, increases in mental health difficulties from Time 1 to Time 2 were associated with poorer social competence. Likewise, gains in caregiver-rated well-being from Time 1 to Time 2 were associated with better social competence. In contrast, initial levels and changes in child-rated well-being were not uniquely associated with social competence at Time 2.[Table tbl3]

## Discussion

Drawing on multi-informant longitudinal ratings of young children’s well-being and social and academic skills, the present study extends the scope of previous work in three ways. First, advancing research beyond middle childhood and adolescence, we used latent variable modeling to show that ratings of children’s well-being and mental health are underpinned by distinct but correlated constructs in the early school years. Second, we found moderate levels of rank-order stability in both child- and caregiver-rated well-being, with remarkably similar levels of stability in mental health. There were declines in average levels of child well-being across informants with a corresponding increase in average levels of caregiver-rated child mental health difficulties. Finally, underscoring the distinct role of well-being and mental health, we found that these constructs exhibited differential associations with social competence and academic performance. Moreover, the relations between well-being and social and academic skills varied by informant, suggesting that both children and caregivers provide distinct insights about children’s well-being.

### Well-Being and Mental Health Are Separable but Related Constructs

The present study sheds new light on the nature of the relation between well-being and mental health. Latent variable models at two separate time points indicated that children’s well-being and mental health were best viewed as distinct but correlated constructs. This was most evident when looking at children’s self-rated well-being and caregiver-rated child mental health. However, even when the same informant (i.e., caregivers) provided information about child well-being and child mental health, the separate factor solution provided the best fit to the data. This suggests that informant effects underpin the stronger correlation between mental health and child well-being in the caregiver data reported here and in the self-report data reported elsewhere (Patalay & Fitzsimons, 2018).

We used bivariate change score models to examine the dynamic relations between well-being and mental health over time. This is the first study to apply this approach to examine the relations between child well-being and mental health. Across informants, these models revealed moderate correlations between changes in well-being and mental health, with declines in well-being being linked to increases in mental health difficulties. While well-being and mental health are separable, our analysis of developmental changes indicated that trajectories for these constructs are associated. This finding has potential importance for screening, as the brevity of the HIFAMS makes it ideally suited for identifying children with negative well-being trajectories that may herald an increase in mental health problems.

Our results highlight the value of adopting a longitudinal approach to investigate changes over time in children’s well-being and mental health. Notably, our study straddled the important but relatively understudied transition from the play-centered pedagogy of the Early Years Foundation Stage to the more formal and academic-focused environment of Key Stage 1 (Fisher, 2010). While some children may embrace the new challenges linked with this transition, the average decline in well-being highlights the need for further studies to investigate the factors that underpin variability in changes in well-being, to guide policy and practice to mitigate the effects of these new challenges on children’s well-being, mental health, and learning (e.g., Rees et al., 2020).

There are at least two possible interpretations for the decline in children’s well-being at the start of formal education. One interpretation is that the decline in well-being is not genuine. According to this interpretation, children’s subjective views of their experience in the first year of primary school may not be reliable (e.g., children’s responses to the questions represent transient states) or valid (e.g., children’s responses to the questions do not signal any meaningful differences between children in their well-being). Indeed, children’s self-reported initial levels of well-being were not related to either social or academic skills 1 year later. Our data challenge the view that young children cannot provide reliable and valid reports about their well-being. Items from the HIFAMS exhibited longitudinal measurement invariance suggesting that children interpreted the items in the same way and with the same degree of reliability on two measurement occasions separated by 12 months. Individual differences in responses from both children and caregivers showed significant stability over time, indicating that children’s reports of well-being do not simply capture transient feelings. Moreover, the latent factor based on children’s ratings was correlated with caregiver ratings of well-being and mental health in the expected directions. The alternative interpretation is that there are genuine declines in young children’s well-being. Declines may be driven by environmental demands (e.g., the shift from the play-based curriculum to more formal academic instruction) or intrinsic factors (e.g., one in three mental health conditions emerge before age 14 suggesting that middle childhood is a particular period of vulnerability) or some combination of these (Fisher, 2010; Solmi et al., 2022). Given that school systems vary in when they move away from play-based early education, cross-country comparisons of children of the same age could be leveraged to disentangle the extent to which changing approaches to education impact children’s well-being.

Further supporting the distinction between mental health and well-being, our longitudinal models revealed that well-being and mental health were linked in distinct ways with children’s social competence and academic abilities in the early school years. For child mental health, *initial levels* (i.e., Reception year SDQ scores) showed unique associations with both social and academic skills 1 year later, which remained significant even when changes in caregiver ratings of child mental health were considered. By contrast, *changes* in both child- and caregiver-rated child well-being (Reception and Year 1) were related to social and academic outcomes, over initial levels of child well-being. Moreover, each of these associations held even when we included important potential confounds (i.e., gender, SES, executive function, language ability, baseline levels of prosocial behavior). That is, rather than showing distinct links with different outcomes (i.e., social and academic domains), mental health and well-being may be distinguished by the *way* they influence outcomes. Specifically, because well-being may show greater change than mental health in the short term (see Figure 3), changes in well-being may matter more than initial levels. Overall, the longitudinal results indicate that the relations between well-being and social and academic skills may be more proximal than those of mental health. In contrast, early mental health difficulties may exert more lasting effects on children’s success at school.

### Different Informants Provide Unique Perspectives on Children’s Well-Being

Research on children’s well-being has largely focused on caregiver reports of young children’s well-being or on self-reported well-being in middle childhood and adolescence (e.g., Patalay & Fitzsimons, 2018). Advances in the measurement of subjective well-being in early childhood (e.g., Allen et al., 2018) provide an opportunity to extend the developmental scope of existing work and may shed light on how children and their caregivers perceive well-being. Specifically, despite using the same questionnaire items, our measurement models revealed that caregiver and child versions of the HIFAMS do not measure the same underlying latent construct (as evident by the poor fit of the model where child- and caregiver-rated items loaded onto one factor). The results suggest that the caregiver and child versions of the HIFAMS questionnaire capture correlated but distinct constructs. Comparison of Time 1 and Time 2 data revealed that caregivers and children’s perspectives on children’s well-being showed an increasing overlap with time but remained distinct. However, the models revealed similar patterns across informants, in terms of both rank-order stability (i.e., moderate stability) and mean-level changes in well-being (i.e., significant declines). Caregivers and children are therefore somewhat aligned in their views about how happy children are at school.

Alongside the similarities noted above, two results from the latent variable models suggest differences in how caregivers and children interpret specific items of the HIFAMS. First, at both time points, measurement models for caregiver-reported child well-being revealed a correlated residual between two items (i.e., “in the playground” and “think about other children”). Correlated residuals indicate that items relate to each other for reasons other than the latent factor (i.e., there is overlapping variance that is not related to well-being; Roos & Bauldry, 2022). By contrast, models of children’s self-reported well-being showed no corresponding correlated residuals between these items. In other words, while children can differentiate their feelings about the playground and about other children, caregivers are more likely to conflate playground activities with peer social interactions. From this, it follows that school initiatives to increase caregivers’ awareness of the highs and lows of children’s playground experiences may be useful, if they enhance caregivers’ abilities to tune into their child’s thoughts and feelings about school.

Second, longitudinal models demonstrated that caregiver- and self-reported well-being had different outcomes. Specifically, gains in caregiver-rated child well-being were linked with children’s social competence but not their academic skills. In contrast, gains in children’s self-reported well-being were linked with their academic skills but not with their social competence. This asymmetry is interesting, as it suggests that children’s feelings about school are more closely related to their experiences of academic success or failure than their caregivers may recognize. That is, for caregivers, perceptions of children’s well-being overlap with perceptions of how children are getting on socially with peers and family members. At a practical level, this finding has potential significance, as it indicates that a useful strategy for increasing caregiver engagement in children’s learning may be to raise caregivers’ awareness of the influence of academic experiences on children’s well-being.

Interestingly, the findings contrast with the results from qualitative research (O’Farrelly et al., 2020) that highlight the importance of children’s friendships for their feelings about school. Unlike the open-ended interviews used in qualitative studies, each item on the HIFAMS prompted children to think about a specific aspect of school (e.g., “When I think about my teacher, I feel …”). The difference in results highlights the need for multimethod studies to enhance our understanding of children’s own views about their school experiences. The results also contrast with findings of unique longitudinal associations between 9- and 10-year-old children’s self-reported happiness and social competence (Yu et al., 2023). These findings further emphasize that patterns of stability and continuity in children’s well-being cannot be generalized from one developmental stage to another. Equally, the between-study contrast in results may highlight differences in the views of children from distinct socioeconomic and cultural backgrounds. That is, while the children in our study were predominantly highly educated and living in England, the children in O’Farrelly et al.’s (2020) qualitative study were from an underresourced community in Ireland while participants in Yu et al.’s (2023) longitudinal study were from China. Further work is needed to elucidate the moderating effects of cultural or demographic factors on the relations between well-being, mental health, and social and academic competence.

### Limitations

Notwithstanding the contributions of the present study, at least four limitations deserve note. First, although our study advances understanding of informant effects on ratings of children’s well-being, we were not able to investigate the overlap between caregiver- and child-rated mental health as existing self-report measures of children’s mental health (e.g., SDQ) were not designed for use with children at the start of primary school (Goodman et al., 2000). Likewise, pandemic restrictions meant that it was only possible to obtain ratings of mental health from children’s teachers at the second study time point. The SDQ exhibits measurement invariance across parents and teachers, indicating that it captures the same underlying constructs with the same degree of reliability (Murray et al., 2021). That said, multiple informants still provide unique insights about contextual differences in the manifestation of children’s mental health. Murray et al. (2021) found that teachers rated children as having fewer mental health difficulties than parents on the SDQ and that teacher and parent ratings were only moderately correlated. Future studies with additional informants (e.g., child and teacher reports of children’s mental health) will illuminate the impact of context on children’s well-being and mental health (Measelle et al., 1998; Stone et al., 2010).

Second, the remote testing protocol used in the present study meant that children completed the measure of subjective well-being with their primary caregiver present. While caregivers completed the assessment of their child’s well-being and mental health prior to this testing session, the presence of caregivers during the children’s assessment may have influenced children’s responses, as children may have presented a more positive perspective on their experiences. That said, the proportions of children rating themselves as “Happy” on each item of the HIFAMS were similar to those reported in an evaluation of the measure, where child assessment was not completed in the presence of a caregiver (Allen et al., 2018).

Third, to capture reliable variance in academic and social skills, we created multimeasure, multi-informant latent factors. These latent factors incorporated caregiver ratings, which may have inflated associations with caregiver-rated child mental health and child well-being. That said, the latent factor for academic skills incorporated objective assessments of reading and numeracy alongside teacher ratings, and the latent factor for social competence included teacher ratings and caregiver ratings. Future studies including more comprehensive objective assessments of children’s academic skills and peer-nomination data will provide a more robust assessment of the impact of child well-being and child mental health on social and academic outcomes, ruling out shared informant effects.

Fourth, pandemic restrictions meant that remote recruitment procedures and testing protocols were used. The use of online recruitment through targeted social media and need to have a stable internet connection and computer with a functioning camera may have limited sample representativeness. The sample was not representative of the U.K. population in terms of education (83% of caregivers had degree-level education compared with 34% in the United Kingdom) or household make-up (e.g., 92% of children in the sample lived in two-parent households compared with 75% in the United Kingdom; Office for National Statistics, 2023, 2024). Furthermore, the samples of Asian children (6% vs. 9%) and Black children (1% vs. 5%) were smaller than expected, while the sample of Mixed or Multiple Ethnic Groups children was larger than expected (13% vs. 5%; Office for National Statistics, 2020). Future work involving more diverse samples of participants is needed to examine the replicability of the findings reported here.

### Conclusions

The current study sheds new light on the relations between children’s well-being and mental health. One key theoretical contribution is that by using multiple informants and multiple time points, converging evidence from our cross-sectional measurement models and longitudinal modeling provided compelling support for the view that well-being and mental health are distinct constructs in young children. The dual-factor account was bolstered by the observation that, even when the same informant reported about children’s mental health and well-being (i.e., caregivers), ratings loaded onto separate latent factors. Furthermore, although mental health and well-being did not neatly predict distinct outcomes in early childhood, the longitudinal models suggested that these constructs differed in the way they related to children’s early social and academic skills. From a practical perspective, our results showed that children’s and caregivers’ ratings of children’s well-being are not interchangeable. Instead, both children and their caregivers provide reliable but unique insights about children’s well-being. Future studies are needed to understand the declines and the wide degree of variation in children’s well-being observed in the early years of primary education.

## Supplementary Material

10.1037/dev0001962.supp

## Figures and Tables

**Table 1 tbl1:** Descriptive Statistics

Measure	T1			T2		
	*M*	*SD*	*N*	*M*	*SD*	*N*
Well-being						
HIFAMS (child)	4.82	1.93	249	4.61	1.97	206
HIFAMS (caregiver)	2.59	2.14	231	2.08	2.09	192
Mental health						
SDQ hyperactivity (caregiver)	3.64	2.44	232	3.66	2.60	192
SDQ conduct problems (caregiver)	1.79	1.63	232	1.82	1.57	192
SDQ emotional problems (caregiver)	1.66	1.78	232	1.96	1.97	192
SDQ peer problems (caregiver)	1.42	1.55	232	1.43	1.52	192
Academic ability						
Word reading (*z*)				0.00	0.99	196
Numeracy screener (*z*)				0.00	0.98	162
Academic competence (caregiver)				5.17	1.38	176
Academic competence (teacher)				4.33	1.46	136
Social adjustment						
SDQ prosocial (caregiver)	7.54	1.93	232	7.76	2.09	192
SDQ prosocial (teacher)				7.97	2.20	154
Peer competence (caregiver)				5.23	1.26	176
SSIS social competence (caregiver)				138.48	14.82	191
PSMS social competence (teacher)				3.58	1.37	133
Covariates						
Receptive vocabulary	27.06	4.07	251			
Backward span task	2.84	1.46	246			
Head toes knees shoulders task	17.20	7.17	246			
Fish flanker task (trials/s)	0.38	0.26	222			
*Note*. T1 = Time 1; T2 = Time 2; HIFAMS = How I Feel About My School; SDQ = Strengths and Difficulties Questionnaire; SSIS = Social Skills Improvement System; PSMS = Peer Social Maturity Scale.						

**Table 2 tbl2:** Model Fit Statistics

Model	Model description	χ^2^	*df*	CFI	TLI	RMSEA
1	Measurement Model A, Time 1	533.252	134	0.671	0.623	0.109
2	Measurement Model B, Time 1	505.516	133	0.692	0.646	0.105
3	Measurement Model C, Time 1	194.746	133	0.949	0.941	0.043
4	Measurement Model D, Time 1	181.280	131	0.959	0.952	0.039
5	Measurement Model A, Time 2	526.351	134	0.761	0.727	0.108
6	Measurement Model B, Time 2	485.138	133	0.786	0.753	0.102
7	Measurement Model C, Time 2	289.765	133	0.905	0.890	0.068
8	Measurement Model D, Time 2	249.697	131	0.928	0.916	0.060
9	Autoregressive model	870.025	598	0.901	0.895	0.042
10	LCS self-rated child well-being	118.819	79	0.960	0.954	0.044
11	LCS caregiver-rated child well-being	147.741	80	0.965	0.961	0.058
12	LCS caregiver-rated child mental health	70.615	25	0.923	0.914	0.085
13	Bivariate LCS self-rated child well-being and mental health	290.469	212	0.931	0.925	0.038
14	Bivariate LCS caregiver-rated child well-being and mental health	405.796	213	0.913	0.906	0.060
15	Caregiver-rated mental health → academic skills	110.626	63	0.923	0.900	0.055
16	Caregiver-rated mental health → social competence	115.870	63	0.922	0.900	0.058
17	Caregiver-rated child well-being → academic skills	271.299	155	0.943	0.930	0.054
18	Caregiver-rated child well-being → social competence	260.000	155	0.951	0.939	0.052
19	Self-rated child well-being → academic skills	230.621	157	0.923	0.906	0.043
20	Self-rated child well-being → social competence	232.151	157	0.922	0.906	0.043
*Note*. CFI = comparative fit index; TLI = Tucker–Lewis index; RMSEA = root-mean-square error of approximation; LCS = latent change score model.						

**Table 3 tbl3:** Standardized Estimates for Models Predicting Academic and Social Skills at Time 2

Variable	Caregiver-rated child mental health		Caregiver-rated child well-being		Self-reported child well-being	
	Academic skill T2	Social skill T2	Academic skill T2	Social skill T2	Academic skill T2	Social skill T2
Mental health initial levels	−.35**	−.45***				
Mental health changes	−.09	−.50***				
Well-being initial levels			.11	.31***	−.03	.09
Well-being changes			.08	.36***	.33***	.04
Gender (0 = girl, 1 = boy)	.02	.03	−.06	−.14*	−.08	−.21***
SES	.04	−.18*	.12*	−.04	.10	−.01
Verbal ability T1	.05	.07	.05	.06	.05	.08
Executive function T1	.29***	−.01	.36***	.03	.29***	.11**
Prosocial behavior T1	−.11	.43***	−.03	.43***	.03	.45***
*Note*. The dependent variable in each model is shown in the first row of the table. T2 = Time 2; SES = socioeconomic status; T1 = Time 1.						
* *p* < .05. ** *p* < .01. *** *p* < .001.						

## References

[ref1] Allen K., Marlow R., Edwards V., Parker C., Rodgers L., Ukoumunne O. C., Seem E. C., Hayes R., Price A., & Ford T. (2018). ‘How I feel about my school’: The construction and validation of a measure of wellbeing at school for primary school children. Clinical Child Psychology and Psychiatry, 23(1), 25–41. 10.1177/1359104516687612 28135832

[ref2] Anwyl-Irvine A. L., Massonnié J., Flitton A., Kirkham N., & Evershed J. K. (2020). Gorilla in our midst: An online behavioral experiment builder. Behavior Research Methods, 52(1), 388–407. 10.3758/s13428-019-01237-x 31016684 PMC7005094

[ref3] Bornstein M. H., Putnick D. L., & Esposito G. (2017). Continuity and stability in development. Child Development Perspectives, 11(2), 113–119. 10.1111/cdep.12221 29503666 PMC5830131

[ref4] Brown T. (2015). Confirmatory factor analysis for applied research (2nd ed.). Guilford Press.

[ref5] Cadman T., Hughes A., Wright C., López-López J. A., Morris T., Rice F., Smith G. D., & Howe L. D. (2021). The role of school enjoyment and connectedness in the association between depressive and externalising symptoms and academic attainment: Findings from a UK prospective cohort study. Journal of Affective Disorders, 295, 974–980. 10.1016/j.jad.2021.08.043 34706471 PMC8572763

[ref6] Cundiff J. M., & Matthews K. A. (2018). Friends with health benefits: The long-term benefits of early peer social integration for blood pressure and obesity in midlife. Psychological Science, 29(5), 814–823. 10.1177/0956797617746510 29533704 PMC5945332

[ref7] Deighton J., Humphrey N., Belsky J., Boehnke J., Vostanis P., & Patalay P. (2018). Longitudinal pathways between mental health difficulties and academic performance during middle childhood and early adolescence. British Journal of Developmental Psychology, 36(1), 110–126. 10.1111/bjdp.12218 29150840

[ref8] Duncan G. J., Dowsett C. J., Claessens A., Magnuson K., Huston A. C., Klebanov P., Pagani L. S., Feinstein L., Engel M., Brooks-Gunn J., Sexton H., Duckworth K., & Japel C. (2007). School readiness and later achievement. Developmental Psychology, 43(6), 1428–1446. 10.1037/0012-1649.43.6.1428 18020822

[ref9] Ferretti F., Chiera A., Nicchiarelli S., Adornetti I., Magni R., Vicari S., Valeri G., & Marini A. (2018). The development of episodic future thinking in middle childhood. Cognitive Processing, 19(1), 87–94. 10.1007/s10339-017-0842-5 29052802

[ref10] Finch W. H., & Bolin J. E. (2017). Multilevel modeling using mplus. Taylor and Francis. 10.1201/9781315165882

[ref11] Fisher J. (2010). Building on the early years foundation stage: Developing good practice for transition into key stage 1. Early Years, 31(1), 31–42. 10.1080/09575146.2010.512557

[ref12] Fivush R. (2011). The development of autobiographical memory. Annual Review of Psychology, 62(1), 559–582. 10.1146/annurev.psych.121208.131702 20636128

[ref13] Ford T., Edwards V., Sharkey S., Ukoumunne O. C., Byford S., Norwich B., & Logan S. (2012). Supporting teachers and children in schools: The effectiveness and cost-effectiveness of the Incredible Years teacher classroom management programme in primary school children: A cluster randomised controlled trial, with parallel economic and process evaluations. BMC Public Health, 12(1), Article 719. 10.1186/1471-2458-12-719 22935476 PMC3488586

[ref14] Goodman R., Ford T., Simmons H., Gatward R., & Meltzer H. (2000). Using the Strengths and Difficulties Questionnaire (SDQ) to screen for child psychiatric disorders in a community sample. The British Journal of Psychiatry, 177(6), 534–539. 10.1192/bjp.177.6.534 11102329

[ref15] Greenspoon P. J., & Saklofske D. H. (2001). Toward an Integration of subjective well-being and psychopathology. Social Indicators Research, 54(1), 81–108. 10.1023/A:1007219227883

[ref16] Gresham F. M., & Elliott S. N. (2008). Social skills improvement system (SSIS). Pearson.

[ref17] Hughes C., Fink E., D’Souza H., & Devine R. T. (2024). The ready or not study: Connecting viewpoints on child and family wellbeing and identifying commonalities across diverse groups, 2021–2023. UK Data Service. 10.5255/UKDA-SN-857098

[ref18] Jirout J. J., Ruzek E., Vitiello V. E., Whittaker J., & Pianta R. C. (2023). The association between and development of school enjoyment and general knowledge. Child Development, 94(2), e119–e127. 10.1111/cdev.13878 36445041

[ref19] Kievit R. A., Brandmaier A. M., Ziegler G., van Harmelen A.-L., de Mooij S. M. M., Moutoussis M., Goodyer I. M., Bullmore E., Jones P. B., Fonagy P., Lindenberger U., Dolan R. J., & the NSPN Consortium. (2018). Developmental cognitive neuroscience using latent change score models: A tutorial and applications. Developmental Cognitive Neuroscience, 33, 99–117. 10.1016/j.dcn.2017.11.007 29325701 PMC6614039

[ref20] Luong R., & Flake J. K. (2023). Measurement invariance testing using confirmatory factor analysis and alignment optimization: A tutorial for transparent analysis planning and reporting. Psychological Methods, 28(4), 905–924. 10.1037/met0000441 35588078

[ref21] Matthews T., Danese A., Caspi A., Fisher H. L., Goldman-Mellor S., Kepa A., Moffitt T. E., Odgers C. L., & Arseneault L. (2019). Lonely young adults in modern Britain: Findings from an epidemiological cohort study. Psychological Medicine, 49(2), 268–277. 10.1017/S0033291718000788 29684289 PMC6076992

[ref22] McNeish D. (2018). Thanks coefficient alpha, we’ll take it from here. Psychological Methods, 23(3), 412–433. 10.1037/met0000144 28557467

[ref23] Measelle J. R., Ablow J. C., Cowan P. A., & Cowan C. P. (1998). Assessing young children’s views of their academic, social, and emotional lives: An evaluation of the self-perception scales of the Berkeley Puppet Interview. Child Development, 69(6), 1556–1576. 10.1111/j.1467-8624.1998.tb06177.x 9914640

[ref24] Mischel W., Shoda Y., & Peake P. K. (1988). The nature of adolescent competencies predicted by preschool delay of gratification. Journal of Personality and Social Psychology, 54(4), 687–696. 10.1037/0022-3514.54.4.687 3367285

[ref25] Morris T. T., Dorling D., Davies N. M., & Davey Smith G. (2021). Associations between school enjoyment at age 6 and later educational achievement: Evidence from a UK cohort study. NPJ Science of Learning, 6(1), Article 18. 10.1038/s41539-021-00092-w 34131153 PMC8206254

[ref26] Murray A. L., Speyer L. G., Hall H. A., Valdebenito S., & Hughes C. (2021). Teacher versus parent informant measurement invariance of the Strengths and Difficulties Questionnaire. Journal of Pediatric Psychology, 46(10), 1249–1257. 10.1093/jpepsy/jsab062 34333621 PMC8561254

[ref27] Muthèn L. K., & Muthèn B. O. (2017). Mplus: Statistical analysis with latent variables. user’s guide (8th ed.).

[ref28] Nosworthy N., Bugden S., Archibald L., Evans B., & Ansari D. (2013). A two-minute paper-and-pencil test of symbolic and nonsymbolic numerical magnitude processing explains variability in primary school children’s arithmetic competence. PLOS ONE, 8(7), Article e67918. 10.1371/journal.pone.0067918 23844126 PMC3699460

[ref29] O’Farrelly C., Booth A., Tatlow-Golden M., & Barker B. (2020). Reconstructing readiness: Young children’s priorities for their early school adjustment. Early Childhood Research Quarterly, 50, 3–16. 10.1016/j.ecresq.2018.12.001

[ref30] Office for National Statistics. (2020). Annual Population Survey: January-December 2020. 10.5255/UKDA-SN-8789-8

[ref31] Office for National Statistics. (2023). Statistical bulletin: Education, England and Wales: Census 2021. https://www.ons.gov.uk/peoplepopulationandcommunity/educationandchildcare/bulletins/educationenglandandwales/census2021

[ref32] Office for National Statistics. (2024). Labour Force Survey (LFS): 1996–2023. https://www.ons.gov.uk/peoplepopulationandcommunity/birthsdeathsandmarriages/families/bulletins/familiesandhouseholds/2023

[ref33] Patalay P., & Fitzsimons E. (2016). Correlates of mental illness and wellbeing in children: Are they the same? Results from the UK millennium cohort study. Journal of the American Academy of Child & Adolescent Psychiatry, 55(9), 771–783. 10.1016/j.jaac.2016.05.019 27566118

[ref34] Patalay P., & Fitzsimons E. (2018). Development and predictors of mental ill-health and wellbeing from childhood to adolescence. Social Psychiatry and Psychiatric Epidemiology, 53(12), 1311–1323. 10.1007/s00127-018-1604-0 30259056

[ref35] Petersen K. J., Humphrey N., & Qualter P. (2020). Latent class analysis of mental health in middle childhood: Evidence for the dual-factor model. School Mental Health, 12(4), 786–800. 10.1007/s12310-020-09384-9

[ref36] Peterson C. C., Slaughter V. P., & Paynter J. (2007). Social maturity and theory of mind in typically developing children and those on the autism spectrum. Journal of Child Psychology and Psychiatry, 48(12), 1243–1250. 10.1111/j.1469-7610.2007.01810.x 18093030

[ref37] Ponitz C. C., McClelland M. M., Matthews J. S., & Morrison F. J. (2009). A structured observation of behavioural self-regulation and its contribution to Kindergarten outcomes. Developmental Psychology, 45(3), 605–619. 10.1037/a0015365 19413419

[ref38] Rees G., Savahl S., Lee B. J., & Casas F. (2020). Children’s views on their lives and well-being in 35 countries: A report on the children’s worlds project, 2016–2019. Children’s Worlds Project. https://isciweb.org/wp-content/uploads/2020/08/Childrens-Worlds-Comparative-Report-2020.pdf

[ref39] Roos M., & Bauldry S. (2022). Confirmatory factor analysis. Sage Publications.

[ref40] Rueda M. R., Fan J., McCandliss B. D., Halparin J. D., Gruber D. B., Lercari L. P., & Posner M. I. (2004). Development of attentional networks in childhood. Neuropsychologia, 42(8), 1029–1040. 10.1016/j.neuropsychologia.2003.12.012 15093142

[ref41] Rust J. (2003). The Wechsler Preschool and Primary Scale of Intelligence (WPPSI-III-UK) (3rd ed.). Harcourt Assessments.

[ref42] Ryan R. M., & Deci E. L. (2001). On happiness and human potentials: A review of research on hedonic and eudaimonic well-being. Annual Review of Psychology, 52(1), 141–166. 10.1146/annurev.psych.52.1.141 11148302

[ref43] Singh-Manoux A., Adler N. E., & Marmot M. G. (2003). Subjective social status: Its determinants and its association with measures of ill-health in the Whitehall II study. Social Science & Medicine, 56(6), 1321–1333. 10.1016/S0277-9536(02)00131-4 12600368

[ref44] Solmi M., Radua J., Olivola M., Croce E., Soardo L., Salazar de Pablo G., Il Shin J., Kirkbride J. B., Jones P., Kim J. H., Kim J. Y., Carvalho A. F., Seeman M. V., Correll C. U., & Fusar-Poli P. (2022). Age at onset of mental disorders worldwide: Large-scale meta-analysis of 192 epidemiological studies. Molecular Psychiatry, 27(1), 281–295. 10.1038/s41380-021-01161-7 34079068 PMC8960395

[ref45] Stone L. L., Otten R., Engels R. C., Vermulst A. A., & Janssens J. M. (2010). Psychometric properties of the parent and teacher versions of the strengths and difficulties questionnaire for 4- to 12-year-olds: A review. Clinical Child and Family Psychology Review, 13(3), 254–274. 10.1007/s10567-010-0071-2 20589428 PMC2919684

[ref46] Suldo S., Thalji A., & Ferron J. (2011). Longitudinal academic outcomes predicted by early adolescents’ subjective well-being, psychopathology, and mental health status yielded from a dual factor model. The Journal of Positive Psychology, 6(1), 17–30. 10.1080/17439760.2010.536774

[ref47] Suldo S. M., & Shaffer E. J. (2008). Looking beyond psychopathology: The dual-factor model of mental health in youth. School Psychology Review, 37(1), 52–68. 10.1080/02796015.2008.12087908

[ref48] Torgesen J. K., Rashotte C. A., & Wagner R. K. (1999). TOWRE: Test of word reading efficiency. Pro-Ed.

[ref49] Woods A. D., Davis-Kean P., Halvorson M., King K., Logan J. R., Xu M., Bainter S., Brown D., Clay J. M., Cruz R. A., Elsherif M. M., Gerasimova D., Joyal-Desmarais K., Moreau D., Nissen J., Schmidt K., Uzdavines A., Van Dusen B., & Vasilev M. (2021). Missing data and multiple imputation decision tree. PsyArXiv. 10.31234/osf.io/mdw5r

[ref50] World Health Organisation. (2022). World mental health report: Transforming mental health for all.

[ref51] Yu Y., Chen X., Li D., Liu J., & Yang F. (2023). Growing up happy: Longitudinal relations between children’s happiness and their social and academic functioning. The Journal of Positive Psychology, 18(4), 531–546. 10.1080/17439760.2022.2093783

