# Supplementary material for: Changes in Children’s Well-Being and Mental Health Across the Early School Years: Links With Academic and Social Competence
Source: Dev Psychol. 2025 Apr 10;61(8):1464–78. doi: 10.1037/dev0001962 (PMC12243392; doi:10.1037/dev0001962)
Supplement: Supplementary file 1 [file DEV-2024-1414_Supplemental_Materials.docx]

**Changes in Children’s Wellbeing and Mental Health Across the Early School Years: Links with Academic and Social Competence**

**Supplementary Materials**

Rory T. Devine^1^, Louise Gray^2^, Miryam Edwards^1^, Mikeda Jess^1^, Caoimhe Dempsey^2^, Jean Heng^2^, Mishika Mehrotra^2^, Hana D’Souza^3^, Elian Fink^4^ & Claire Hughes^2^

^1^University of Birmingham, Center for Developmental Science, United Kingdom

^2^University of Cambridge, Center for Family Research, United Kingdom

^3^Cardiff University, School of Psychology, United Kingdom

^4^University of Sussex, School of Psychology, United Kingdom

**Author Note.** The project was funded through the Economic and Social Research Council, UK (ES/T016175/1). Correspondence can be sent to: [R.T.Devine@bham.ac.uk](mailto:R.T.Devine@bham.ac.uk). The analyses were not pre-registered. The analytic code is in the Supplementary Materials. Data are available from the UK Data Service: https://dx.doi.org/10.5255/UKDA-SN-857098.

**Sample Size Justification**

The initial plan for the current study was to track a pre-existing cohort of 200 children from the United Kingdom across the transition from Reception Year to Year 1 and to recruit an additional 250 children to enrich the sample. Restrictions due to the Covid-19 pandemic in 2020 and 2021 meant that the start date for the project was delayed and the opportunity to collect data from the pre-existing cohort was no longer possible as the cohort children had moved beyond Reception Year. We instead recruited a new sample of children for the current study. Monte Carlo simulations with 1000 replications in *Mplus* (Muthèn & Muthèn, 2017) indicated that a sample size of 250 children would provide sufficient (.81 - .85) power to detect moderate-to-strong cross-lagged effects between two latent variables in a longitudinal model of two time points with three covariates.

**Data Reduction**

We sought to create multi-measure, multi-informant scores for children’s academic skills and social competence. We used confirmatory factor analysis (CFA) with a robust maximum likelihood (MLR) estimator to test two models. First, we tested a model where children’s scores for reading and numeracy, teacher ratings of academic performance, and parent-rated academic skills loaded onto a single latent factor. The model provided a good fit to the data, $\chi^{2}\left( 2 \right)=0.141, p=0.93, CFI=1.00, TLI=1.05, RMSEA=0$. All indicators loaded significantly onto the latent factor and the factor accounted for between 13% and 78% of the variance in the four indicators. Next, we tested a model where parent-rated social competence, peer relationships, and prosocial behavior loaded onto a latent factor with teacher-rated social maturity and prosocial behavior. We permitted the residuals for the two teacher indicators to covary to account for informant effects. The model provided an acceptable fit to the data, $\chi^{2}\left( 4 \right)=10.06, p=0.04, CFI=0.97, TLI=0.93, RMSEA=0.08.$ All indicators loaded significantly onto the latent factor and the factor accounted for between 19% and 67% of the variance in each indicator. We used the latent factor scores for academic skills and social competence in subsequent models. We estimated latent factor scores for academic skills for 220 children and social competence for 216 children.

We also used CFA to test a one-factor model of executive function. We used MLR estimation to test a model where scores from the Backward Animal Span, Fish Flanker Task and Head Toes Knees Shoulders Task loaded onto a single latent factor. The model was just identified with 0 degrees of freedom so there were no model fit indices (Roos & Bauldry, 2022). All loadings were statistically significant, all *p*s <0.0001, and the latent factor explained between 17% – 33% of the variance in each indicator. We estimated the factor scores for the executive function latent factor and used these in subsequent analyses.

**Correlates of Missingness**

*Table S1. Results of Logistic Regression Showing Time 1 Correlates of Missing Data.*

|  | **Participation at T1 and T2** | | |  | **Teacher Questionnaire at T2** | | |
| --- | --- | --- | --- | --- | --- | --- | --- |
|  | ***Est.*** | ***OR*** | ***p*** |  | ***Est.*** | ***OR*** | ***p*** |
| Gender | -0.129 | 0.879 | 0.748 |  | -0.246 | 0.782 | 0.364 |
| Age | -1.266 | 0.282 | 0.062 |  | -0.659 | 0.517 | 0.120 |
| SES | -0.037 | 0.963 | 0.853 |  | 0.151 | 1.163 | 0.348 |
| Child Wellbeing | -0.074 | 0.929 | 0.528 |  | -0.010 | 0.990 | 0.891 |
| Verbal Ability | 0.264 | 1.303 | 0.179 |  | 0.107 | 1.113 | 0.411 |

*Note.* Est. = Robust Maximum Likelihood Estimate. OR = Odds Ratio. Child Wellbeing = Summed Score for Child-Rated HIFAMS Questionnaire. Participation at T1 and T2 was dummy coded as 0 (T1 only) and 1 (T1 and T2). Teacher Questionnaire at T2 was dummy coded as 0 (no questionnaire) and 1 (questionnaire returned).

**Longitudinal Measurement Invariance for Child Wellbeing and Mental Health**

We used nested model comparisons to test for longitudinal measurement invariance in self-reported child wellbeing, caregiver-reported child wellbeing, and caregiver-reported child mental health (Luong & Flake, 2023). For each construct, we first tested for configural invariance by specifying the same measurement model at Time 1 and Time 2. We freely estimated the latent factor means by fixing the threshold (or intercept for continuous indicators) at 0. We also fixed the first loading of each factor to 1 and freely estimated the latent factor variances at each time point. We permitted like indicator residuals to correlate over time and allowed the Time 1 latent factor to correlate with the Time 2 latent factor. To test for weak factorial invariance, we extended this first model by constraining factor loadings to equality across time. Finally, we tested for strong factorial invariance by constraining indicator thresholds (for categorical items) and intercepts (for continuous items) to equality. The results of measurement invariance testing procedures are shown in Table S2.

*Table S2. Model Comparisons for Measurement Invariance Testing*

|  | $\boldsymbol{\chi}^{\boldsymbol{2}}$ | ***df*** | **CFI** | **TLI** | **RMSEA** | $\boldsymbol{\Delta\chi}^{\boldsymbol{2}}$ | ***df*** | ***ΔCFI*** | ***ΔRMSEA*** |
| --- | --- | --- | --- | --- | --- | --- | --- | --- | --- |
| **Child HIFAMS** | | |  |  |  |  |  |  |  |
| Configural | 86.95 | 69 | 0.977 | 0.970 | 0.032 | - | - |  |  |
| Weak | 88.52 | 75 | 0.983 | 0.979 | 0.027 | 5.178 | 6 | +0.006 | -0.005 |
| Strong | 107.33 | 81 | 0.966 | 0.962 | 0.036 | 25.17* | 6 | -0.017 | +0.009 |
| Partial | 97.21 | 79 | 0.977 | 0.973 | 0.030 | 11.26* | 4 | +0.006 | -0.006 |
| **Caregiver HIFAMS** | | |  |  |  |  |  |  |  |
| Configural | 81.33 | 67 | 0.991 | 0.988 | 0.030 | - | - |  |  |
| Weak | 92.49 | 73 | 0.988 | 0.984 | 0.033 | 10.14 | 6 | -0.003 | +0.003 |
| Strong | 100.17 | 79 | 0.986 | 0.984 | 0.033 | 8.42 | 6 | -0.002 | 0 |
| Covariance | 101.56 | 80 | 0.986 | 0.984 | 0.033 | 1.62 | 1 | 0 | 0 |
| **Caregiver SDQ** | | |  |  |  |  |  |  |  |
| Configural | 37.29 | 15 | 0.948 | 0.903 | 0.078 | - | - |  |  |
| Weak | 37.21 | 18 | 0.955 | 0.930 | 0.066 | 1.88 | 3 | +0.007 | -0.012 |
| Strong | 46.75 | 21 | 0.940 | 0.920 | 0.071 | 10.16* | 3 | -0.015 | +0.005 |
| Residuals | 52.92 | 25 | 0.935 | 0.927 | 0.068 | 6.14 | 4 | -0.005 | -0.003 |

*Note.* **p* < .05. HIFAMS = How I Feel About My School Questionnaire. SDQ = Strengths and Difficulties Questionnaire. Declines in model fit were identified using three criteria: a significant increase in $\chi^{2}$, a decrease in CFI of $\geq0.01$, and an increase in RMSEA of $\geq0.015.$Two thresholds (‘when I’m in the classroom’ and ‘when I think about my teacher’) were freely estimated in the Partial Model for the Child-Rated HIFAMS. The WLSMV $\chi^{2}$difference test was used to compare the Child HIFAMS and Parent HIFAMS models. The Satorra Bentler scaled $\chi^{2}$ difference test was used for the Parent SDQ model.

Table S3. *Internal Consistency Reliability of Questionnaire Measures (Cronbach’s Alpha)*

| **Measure** | **Time 1** | **Time 2** |
| --- | --- | --- |
| Caregiver SDQ Hyperactivity | .79 | .81 |
| Caregiver SDQ Conduct Problems | .60 | .61 |
| Caregiver SDQ Emotional Problems | .68 | .69 |
| Caregiver SDQ Peer Problems | .57 | .59 |
| Caregiver SDQ Prosocial Behavior | .71 | .80 |
| Teacher SDQ Prosocial Behavior |  | .81 |
| Caregiver SSIS Total |  | .86 |
| Teacher Peer Social Maturity Scale |  | .97 |
| Teacher Assessment of Academic Ability |  | .97 |

**Data Analysis Code (Mplus)**

**Missing Data Analysis**

!Variables predicting participation at T1 and T2

Variable:

Names are

ID !Family ID number

T2PART !0 = T1 only, 1 = T1 and T2

T2CGEND !0=Girl 1=Boy

T1CAGEY !Age in years

SESZ !Family socio-economic status

T1CHIFAM !Child Self-Reported Wellbeing T1

T1VAZ !T1 Verbal Ability

Usevar are T2PART T2CGEND T1CAGEY SESZ T1CHIFAM T1VAZ;

Categorical = T2PART;

Missing are all (-99);

IDVAR = ID;

Analysis:

Estimator = MLR;

Integration = Montecarlo;

Model:

T2PART ON T2CGEND T1CAGEY SESZ T1CHIFAM T1VAZ;

[T1CAGEY]; [SESZ]; [T1CHIFAM]; [T1VAZ];

Output: Standardized;

!Variables predicting Teacher Questionnaire at Time 2

Variable:

Names are

ID !Family ID number

T2TEACH !0 = no questionnaire, 1 = questionnaire returned

T2CGEND !0=Girl 1=Boy

T1CAGEY !Age in years

SESZ !Family socio-economic status

T1CHIFAM !Child Self-Reported Wellbeing T1

T1VAZ !T1 Verbal Ability

Usevar are T2TEACH T2CGEND T1CAGEY SESZ T1CHIFAM T1VAZ;

Categorical = T2TEACH

Missing are all (-99);

IDVAR = ID;

Analysis:

Estimator = MLR;

Integration = Montecarlo;

Model:

T2TEACH ON T2CGEND T1CAGEY SESZ T1CHIFAM T1VAZ;

[T1CAGEY]; [SESZ]; [T1CHIFAM]; [T1VAZ];

Output: Standardized;

**Multiple Imputation**

**Variable:** Names are ID

T2CGEND !0=girl 1=boy

T1CAGEY !age in years

T1CHIFB1 !T1 Child self-report HIFAMS

T1CHIFB2 T1CHIFB3 T1CHIFB4 T1CHIFB5 T1CHIFB6 T1CHIFB7

T2CHIFB1 !T2 Child self-report HIFAMS

T2CHIFB2 T2CHIFB3 T2CHIFB4 T2CHIFB5 T2CHIFB6 T2CHIFB7

T1PHIFB1 !T1 Caregiver-report HIFAMS

T1PHIFB2 T1PHIFB3 T1PHIFB4 T1PHIFB5 T1PHIFB6 T1PHIFB7

T2PHIFB1 !T2 Caregiver-report HIFAMS

T2PHIFB2 T2PHIFB3 T2PHIFB4 T2PHIFB5 T2PHIFB6 T2PHIFB7

T1PSDPRO !T1 Caregiver SDQ Prosocial

T1PSDHYP !T1 Caregiver SDQ Hyperactivity

T1PSDEMO !T1 Caregiver SDQ Emotional Problems

T1PSDCON !T1 Caregiver SDQ Conduct Problems

T1PSDPPR !T1 Caregiver SDQ Peer Problems

T2PSDHYP !T2 Caregiver SDQ Hyperactivity

T2PSDEMO !T2 Caregiver SDQ Emotional Problems

T2PSDCON !T2 Caregiver SDQ Conduct Problems

T2PSDPPR !T2 Caregiver SDQ Peer Problems

T1VAZ !T1 Verbal Ability

SESZ !SES

T1EFFS !T1 EF Latent Factor Scores

T2ACADFS !T2 Academic Skills Latent Factor Scores

T2SOCFS; !T2 Social Competence Latent Factor Scores

Usevar are T2CGEND T1CAGEY T1CHIFB1 T1CHIFB2 T1CHIFB3 T1CHIFB4 T1CHIFB5 T1CHIFB6 T1CHIFB7 T2CHIFB1 T2CHIFB2 T2CHIFB3 T2CHIFB4 T2CHIFB5 T2CHIFB6 T2CHIFB7 T1PHIFB1 T1PHIFB2 T1PHIFB3 T1PHIFB4 T1PHIFB5 T1PHIFB6 T1PHIFB7 T2PHIFB1 T2PHIFB2 T2PHIFB3 T2PHIFB4 T2PHIFB5 T2PHIFB6 T2PHIFB7 T1PSDPRO T1PSDHYP T1PSDEMO T1PSDCON T1PSDPPR T2PSDHYP T2PSDEMO T2PSDCON T2PSDPPR T1VAZ SESZ T1EFFS T2ACADFS T2SOCFS;

Categorical = T2CGEND T1CHIFB1 T1CHIFB2 T1CHIFB3 T1CHIFB4 T1CHIFB5 T1CHIFB6 T1CHIFB7 T2CHIFB1 T2CHIFB2 T2CHIFB3 T2CHIFB4 T2CHIFB5 T2CHIFB6 T2CHIFB7 T1PHIFB1 T1PHIFB2 T1PHIFB3 T1PHIFB4 T1PHIFB5 T1PHIFB6 T1PHIFB7 T2PHIFB1 T2PHIFB2 T2PHIFB3 T2PHIFB4 T2PHIFB5 T2PHIFB6 T2PHIFB7 T2THIFB1 T2THIFB2 T2THIFB3 T2THIFB4;

Missing are all (-99); IDVAR = ID;

**Data Imputation:**

Impute =T2CGEND (c) T1CHIFB1 (c) T1CHIFB2 (c) T1CHIFB3 (c) T1CHIFB4 (c) T1CHIFB5 (c) T1CHIFB6 (c) T1CHIFB7 (c) T2CHIFB1 (c) T2CHIFB2 (c) T2CHIFB3 (c)

T2CHIFB4 (c) T2CHIFB5 (c) T2CHIFB6 (c) T2CHIFB7 (c) T1PHIFB1 (c) T1PHIFB2 (c) T1PHIFB3 (c) T1PHIFB4 (c) T1PHIFB5 (c) T1PHIFB6 (c) T1PHIFB7 (c) T2PHIFB1 (c) T2PHIFB2 (c) T2PHIFB3 (c) T2PHIFB4 (c) T2PHIFB5 (c) T2PHIFB6 (c) T2PHIFB7 (c) T1CAGEY T1PSDPRO T1PSDHYP T1PSDEMO T1PSDCON T1PSDPPR T2PSDHYP T2PSDEMO T2PSDCON T2PSDPPR T1VAZ SESZ T1EFFS T2ACADFS T2SOCFS;

Ndatasets = 50; Save = RoNWB*.dat;

**Analysis:** Type = basic; Bseed = 12345;

**Output:** Tech8;

**Measurement Models**

**Model 1: Model A, Time 1: Single Factor Model**

**Data:**

File = RoNWBlist.dat;

Type = Imputation;

**Variable:** Names are T2CGEND T1CAGEY T1CHIFB1 T1CHIFB2 T1CHIFB3 T1CHIFB4 T1CHIFB5 T1CHIFB6 T1CHIFB7 T2CHIFB1 T2CHIFB2 T2CHIFB3 T2CHIFB4 T2CHIFB5 T2CHIFB6 T2CHIFB7 T1PHIFB1 T1PHIFB2 T1PHIFB3 T1PHIFB4 T1PHIFB5 T1PHIFB6 T1PHIFB7 T2PHIFB1 T2PHIFB2 T2PHIFB3 T2PHIFB4 T2PHIFB5 T2PHIFB6 T2PHIFB7 T2THIFB1 T2THIFB2 T2THIFB3 T2THIFB4 T1PSDPRO T1PSDHYP T1PSDEMO T1PSDCON T1PSDPPR T2PSDHYP T2PSDEMO T2PSDCON T2PSDPPR T1VAZ SESZ T1EFFS T2ACADFS T2SOCFS ID;

Usevar are T1CHIFB1 T1CHIFB2 T1CHIFB3 T1CHIFB4 T1CHIFB5 T1CHIFB6 T1CHIFB7 T1PHIFB1 T1PHIFB2 T1PHIFB3 T1PHIFB4 T1PHIFB5 T1PHIFB6 T1PHIFB7 T1PSDHYP T1PSDEMO T1PSDCON T1PSDPPR;

Categorical = T1CHIFB1 T1CHIFB2 T1CHIFB3 T1CHIFB4 T1CHIFB5 T1CHIFB6 T1CHIFB7 T1PHIFB1 T1PHIFB2 T1PHIFB3 T1PHIFB4 T1PHIFB5 T1PHIFB6 T1PHIFB7;

Missing are all (-99); IDVAR = ID;

**Analysis:** Estimator = WLSMV;

Model: WB BY T1CHIFB1 T1CHIFB2 T1CHIFB3 T1CHIFB4 T1CHIFB5 T1CHIFB6 T1CHIFB7 T1PSDHYP T1PSDEMO T1PSDCON T1PSDPPR T1PHIFB1 T1PHIFB2 T1PHIFB3 T1PHIFB4 T1PHIFB5 T1PHIFB6 T1PHIFB7;

T1PHIFB4 with T1PHIFB5;

**Output:** Sampstat; Standardized;

**Model 2: Model B, Time 1: Dual Factor Model (Wellbeing and Mental Health)**

**Data:**

File = RoNWBlist.dat;

Type = Imputation;

**Variable:** Names are T2CGEND T1CAGEY T1CHIFB1 T1CHIFB2 T1CHIFB3 T1CHIFB4 T1CHIFB5 T1CHIFB6 T1CHIFB7 T2CHIFB1 T2CHIFB2 T2CHIFB3 T2CHIFB4 T2CHIFB5 T2CHIFB6 T2CHIFB7 T1PHIFB1 T1PHIFB2 T1PHIFB3 T1PHIFB4 T1PHIFB5 T1PHIFB6 T1PHIFB7 T2PHIFB1 T2PHIFB2 T2PHIFB3 T2PHIFB4 T2PHIFB5 T2PHIFB6 T2PHIFB7 T2THIFB1 T2THIFB2 T2THIFB3 T2THIFB4 T1PSDPRO T1PSDHYP T1PSDEMO T1PSDCON T1PSDPPR T2PSDHYP T2PSDEMO T2PSDCON T2PSDPPR T1VAZ SESZ T1EFFS T2ACADFS T2SOCFS ID;

Usevar are T1CHIFB1 T1CHIFB2 T1CHIFB3 T1CHIFB4 T1CHIFB5 T1CHIFB6 T1CHIFB7 T1PHIFB1 T1PHIFB2 T1PHIFB3 T1PHIFB4 T1PHIFB5 T1PHIFB6 T1PHIFB7 T1PSDHYP T1PSDEMO T1PSDCON T1PSDPPR;

Categorical = T1CHIFB1 T1CHIFB2 T1CHIFB3 T1CHIFB4 T1CHIFB5 T1CHIFB6 T1CHIFB7 T1PHIFB1 T1PHIFB2 T1PHIFB3 T1PHIFB4 T1PHIFB5 T1PHIFB6 T1PHIFB7;

Missing are all (-99); IDVAR = ID;

**Analysis:** Estimator = WLSMV;

**Model:**

WB BY T1CHIFB1 T1CHIFB2 T1CHIFB3 T1CHIFB4 T1CHIFB5 T1CHIFB6 T1CHIFB7 T1PHIFB1 T1PHIFB2 T1PHIFB3 T1PHIFB4 T1PHIFB5 T1PHIFB6 T1PHIFB7;

T1PHIFB4 with T1PHIFB5;

MH BY T1PSDHYP T1PSDEMO T1PSDCON T1PSDPPR;

WB with MH;

**Output:** Sampstat; Standardized;

**Model 3: Model C, Time 1: Dual Factor Model (Caregiver and Child Informant)**

**Data:**

File = RoNWBlist.dat;

Type = Imputation;

**Variable:** Names are T2CGEND T1CAGEY T1CHIFB1 T1CHIFB2 T1CHIFB3 T1CHIFB4 T1CHIFB5 T1CHIFB6 T1CHIFB7 T2CHIFB1 T2CHIFB2 T2CHIFB3 T2CHIFB4 T2CHIFB5 T2CHIFB6 T2CHIFB7 T1PHIFB1 T1PHIFB2 T1PHIFB3 T1PHIFB4 T1PHIFB5 T1PHIFB6 T1PHIFB7 T2PHIFB1 T2PHIFB2 T2PHIFB3 T2PHIFB4 T2PHIFB5 T2PHIFB6 T2PHIFB7 T2THIFB1 T2THIFB2 T2THIFB3 T2THIFB4 T1PSDPRO T1PSDHYP T1PSDEMO T1PSDCON T1PSDPPR T2PSDHYP T2PSDEMO T2PSDCON T2PSDPPR T1VAZ SESZ T1EFFS T2ACADFS T2SOCFS ID;

Usevar are T1CHIFB1 T1CHIFB2 T1CHIFB3 T1CHIFB4 T1CHIFB5 T1CHIFB6 T1CHIFB7 T1PHIFB1 T1PHIFB2 T1PHIFB3 T1PHIFB4 T1PHIFB5 T1PHIFB6 T1PHIFB7 T1PSDHYP T1PSDEMO T1PSDCON T1PSDPPR;

Categorical = T1CHIFB1 T1CHIFB2 T1CHIFB3 T1CHIFB4 T1CHIFB5 T1CHIFB6 T1CHIFB7 T1PHIFB1 T1PHIFB2 T1PHIFB3 T1PHIFB4 T1PHIFB5 T1PHIFB6 T1PHIFB7;

Missing are all (-99); IDVAR = ID;

**Analysis:** Estimator = WLSMV;

**Model:**

CHILD BY T1CHIFB1 T1CHIFB2 T1CHIFB3 T1CHIFB4 T1CHIFB5 T1CHIFB6 T1CHIFB7;

CARER BY T1PSDHYP T1PSDEMO T1PSDCON T1PSDPPR T1PHIFB1 T1PHIFB2 T1PHIFB3 T1PHIFB4 T1PHIFB5 T1PHIFB6 T1PHIFB7;

T1PHIFB4 with T1PHIFB5;

CHILD with CARER;

**Output:** Sampstat; Standardized;

**Model 4: Model D, Time 1: Three Factor Model**

**Data:**

File = RoNWBlist.dat;

Type = Imputation;

**Variable:** Names are T2CGEND T1CAGEY T1CHIFB1 T1CHIFB2 T1CHIFB3 T1CHIFB4 T1CHIFB5 T1CHIFB6 T1CHIFB7 T2CHIFB1 T2CHIFB2 T2CHIFB3 T2CHIFB4 T2CHIFB5 T2CHIFB6 T2CHIFB7 T1PHIFB1 T1PHIFB2 T1PHIFB3 T1PHIFB4 T1PHIFB5 T1PHIFB6 T1PHIFB7 T2PHIFB1 T2PHIFB2 T2PHIFB3 T2PHIFB4 T2PHIFB5 T2PHIFB6 T2PHIFB7 T2THIFB1 T2THIFB2 T2THIFB3 T2THIFB4 T1PSDPRO T1PSDHYP T1PSDEMO T1PSDCON T1PSDPPR T2PSDHYP T2PSDEMO T2PSDCON T2PSDPPR T1VAZ SESZ T1EFFS T2ACADFS T2SOCFS ID;

Usevar are T1CHIFB1 T1CHIFB2 T1CHIFB3 T1CHIFB4 T1CHIFB5 T1CHIFB6 T1CHIFB7 T1PHIFB1 T1PHIFB2 T1PHIFB3 T1PHIFB4 T1PHIFB5 T1PHIFB6 T1PHIFB7 T1PSDHYP T1PSDEMO T1PSDCON T1PSDPPR;

Categorical = T1CHIFB1 T1CHIFB2 T1CHIFB3 T1CHIFB4 T1CHIFB5 T1CHIFB6 T1CHIFB7 T1PHIFB1 T1PHIFB2 T1PHIFB3 T1PHIFB4 T1PHIFB5 T1PHIFB6 T1PHIFB7;

Missing are all (-99); IDVAR = ID;

**Analysis:** Estimator = WLSMV;

**Model:**

CWB BY T1CHIFB1 T1CHIFB2 T1CHIFB3 T1CHIFB4 T1CHIFB5 T1CHIFB6 T1CHIFB7;

CGWB BY T1PHIFB1 T1PHIFB2 T1PHIFB3 T1PHIFB4 T1PHIFB5 T1PHIFB6 T1PHIFB7; T1PHIFB4 with T1PHIFB5;

CGMH BY T1PSDHYP T1PSDEMO T1PSDCON T1PSDPPR;

CWB with CGWB CGMH; CGWB with CGMH;

**Output:** Sampstat; Standardized;

**Model 5: Model A, Time 2: Single Factor Model**

**Data:**

File = RoNWBlist.dat;

Type = Imputation;

**Variable:** Names are T2CGEND T1CAGEY T1CHIFB1 T1CHIFB2 T1CHIFB3 T1CHIFB4 T1CHIFB5 T1CHIFB6 T1CHIFB7 T2CHIFB1 T2CHIFB2 T2CHIFB3 T2CHIFB4 T2CHIFB5 T2CHIFB6 T2CHIFB7 T1PHIFB1 T1PHIFB2 T1PHIFB3 T1PHIFB4 T1PHIFB5 T1PHIFB6 T1PHIFB7 T2PHIFB1 T2PHIFB2 T2PHIFB3 T2PHIFB4 T2PHIFB5 T2PHIFB6 T2PHIFB7 T2THIFB1 T2THIFB2 T2THIFB3 T2THIFB4 T1PSDPRO T1PSDHYP T1PSDEMO T1PSDCON T1PSDPPR T2PSDHYP T2PSDEMO T2PSDCON T2PSDPPR T1VAZ SESZ T1EFFS T2ACADFS T2SOCFS ID;

Usevar are T2CHIFB1 T2CHIFB2 T2CHIFB3 T2CHIFB4 T2CHIFB5 T2CHIFB6 T2CHIFB7 T2PHIFB1 T2PHIFB2 T2PHIFB3 T2PHIFB4 T2PHIFB5 T2PHIFB6 T2PHIFB7 T2PSDHYP T2PSDEMO T2PSDCON T2PSDPPR;

Categorical = T2CHIFB1 T2CHIFB2 T2CHIFB3 T2CHIFB4 T2CHIFB5 T2CHIFB6 T2CHIFB7 T2PHIFB1 T2PHIFB2 T2PHIFB3 T2PHIFB4 T2PHIFB5 T2PHIFB6 T2PHIFB7;

Missing are all (-99); IDVAR = ID;

**Analysis:** Estimator = WLSMV;

Model: WB BY T2CHIFB1 T2CHIFB2 T2CHIFB3 T2CHIFB4 T2CHIFB5 T2CHIFB6 T2CHIFB7 T2PHIFB1 T2PHIFB2 T2PHIFB3 T2PHIFB4 T2PHIFB5 T2PHIFB6 T2PHIFB7 T2PSDHYP T2PSDEMO T2PSDCON T2PSDPPR;

T2PHIFB4 with T2PHIFB5;

**Output:** Sampstat; Standardized;

**Model 6: Model B, Time 2: Dual Factor Model (Wellbeing and Mental Health)**

**Data:**

File = RoNWBlist.dat;

Type = Imputation;

**Variable:** Names are T2CGEND T1CAGEY T1CHIFB1 T1CHIFB2 T1CHIFB3 T1CHIFB4 T1CHIFB5 T1CHIFB6 T1CHIFB7 T2CHIFB1 T2CHIFB2 T2CHIFB3 T2CHIFB4 T2CHIFB5 T2CHIFB6 T2CHIFB7 T1PHIFB1 T1PHIFB2 T1PHIFB3 T1PHIFB4 T1PHIFB5 T1PHIFB6 T1PHIFB7 T2PHIFB1 T2PHIFB2 T2PHIFB3 T2PHIFB4 T2PHIFB5 T2PHIFB6 T2PHIFB7 T2THIFB1 T2THIFB2 T2THIFB3 T2THIFB4 T1PSDPRO T1PSDHYP T1PSDEMO T1PSDCON T1PSDPPR T2PSDHYP T2PSDEMO T2PSDCON T2PSDPPR T1VAZ SESZ T1EFFS T2ACADFS T2SOCFS ID;

Usevar are T2CHIFB1 T2CHIFB2 T2CHIFB3 T2CHIFB4 T2CHIFB5 T2CHIFB6 T2CHIFB7 T2PHIFB1 T2PHIFB2 T2PHIFB3 T2PHIFB4 T2PHIFB5 T2PHIFB6 T2PHIFB7 T2PSDHYP T2PSDEMO T2PSDCON T2PSDPPR;

Categorical = T2CHIFB1 T2CHIFB2 T2CHIFB3 T2CHIFB4 T2CHIFB5 T2CHIFB6 T2CHIFB7 T2PHIFB1 T2PHIFB2 T2PHIFB3 T2PHIFB4 T2PHIFB5 T2PHIFB6 T2PHIFB7;

Missing are all (-99); IDVAR = ID;

**Analysis:** Estimator = WLSMV;

Model:

WB BY T2CHIFB1 T2CHIFB2 T2CHIFB3 T2CHIFB4 T2CHIFB5 T2CHIFB6 T2CHIFB7 T2PHIFB1 T2PHIFB2 T2PHIFB3 T2PHIFB4 T2PHIFB5 T2PHIFB6 T2PHIFB7;

T2PHIFB4 with T2PHIFB5;

MH BY T2PSDHYP T2PSDEMO T2PSDCON T2PSDPPR; MH with WB;

**Output:** Sampstat; Standardized;

**Model 7: Model C, Time 2: Dual Factor Model (Caregiver and Child Informant)**

**Data:**

File = RoNWBlist.dat;

Type = Imputation;

**Variable:** Names are T2CGEND T1CAGEY T1CHIFB1 T1CHIFB2 T1CHIFB3 T1CHIFB4 T1CHIFB5 T1CHIFB6 T1CHIFB7 T2CHIFB1 T2CHIFB2 T2CHIFB3 T2CHIFB4 T2CHIFB5 T2CHIFB6 T2CHIFB7 T1PHIFB1 T1PHIFB2 T1PHIFB3 T1PHIFB4 T1PHIFB5 T1PHIFB6 T1PHIFB7 T2PHIFB1 T2PHIFB2 T2PHIFB3 T2PHIFB4 T2PHIFB5 T2PHIFB6 T2PHIFB7 T2THIFB1 T2THIFB2 T2THIFB3 T2THIFB4 T1PSDPRO T1PSDHYP T1PSDEMO T1PSDCON T1PSDPPR T2PSDHYP T2PSDEMO T2PSDCON T2PSDPPR T1VAZ SESZ T1EFFS T2ACADFS T2SOCFS ID;

Usevar are T2CHIFB1 T2CHIFB2 T2CHIFB3 T2CHIFB4 T2CHIFB5 T2CHIFB6 T2CHIFB7 T2PHIFB1 T2PHIFB2 T2PHIFB3 T2PHIFB4 T2PHIFB5 T2PHIFB6 T2PHIFB7 T2PSDHYP T2PSDEMO T2PSDCON T2PSDPPR;

Categorical = T2CHIFB1 T2CHIFB2 T2CHIFB3 T2CHIFB4 T2CHIFB5 T2CHIFB6 T2CHIFB7 T2PHIFB1 T2PHIFB2 T2PHIFB3 T2PHIFB4 T2PHIFB5 T2PHIFB6 T2PHIFB7;

Missing are all (-99); IDVAR = ID;

**Analysis:** Estimator = WLSMV;

Model:

CHILD BY T2CHIFB1 T2CHIFB2 T2CHIFB3 T2CHIFB4 T2CHIFB5 T2CHIFB6 T2CHIFB7;

CARER BY T2PHIFB1 T2PHIFB2 T2PHIFB3 T2PHIFB4 T2PHIFB5 T2PHIFB6 T2PHIFB7 T2PSDHYP T2PSDEMO T2PSDCON T2PSDPPR; T2PHIFB4 with T2PHIFB5;

CHILD with CARER;

**Output:** Sampstat; Standardized;

**Model 8: Model D, Time 2: Three Factor Model**

**Data:**

File = RoNWBlist.dat;

Type = Imputation;

**Variable:** Names are T2CGEND T1CAGEY T1CHIFB1 T1CHIFB2 T1CHIFB3 T1CHIFB4 T1CHIFB5 T1CHIFB6 T1CHIFB7 T2CHIFB1 T2CHIFB2 T2CHIFB3 T2CHIFB4 T2CHIFB5 T2CHIFB6 T2CHIFB7 T1PHIFB1 T1PHIFB2 T1PHIFB3 T1PHIFB4 T1PHIFB5 T1PHIFB6 T1PHIFB7 T2PHIFB1 T2PHIFB2 T2PHIFB3 T2PHIFB4 T2PHIFB5 T2PHIFB6 T2PHIFB7 T2THIFB1 T2THIFB2 T2THIFB3 T2THIFB4 T1PSDPRO T1PSDHYP T1PSDEMO T1PSDCON T1PSDPPR T2PSDHYP T2PSDEMO T2PSDCON T2PSDPPR T1VAZ SESZ T1EFFS T2ACADFS T2SOCFS ID;

Usevar are T2CHIFB1 T2CHIFB2 T2CHIFB3 T2CHIFB4 T2CHIFB5 T2CHIFB6 T2CHIFB7 T2PHIFB1 T2PHIFB2 T2PHIFB3 T2PHIFB4 T2PHIFB5 T2PHIFB6 T2PHIFB7 T2PSDHYP T2PSDEMO T2PSDCON T2PSDPPR;

Categorical = T2CHIFB1 T2CHIFB2 T2CHIFB3 T2CHIFB4 T2CHIFB5 T2CHIFB6 T2CHIFB7 T2PHIFB1 T2PHIFB2 T2PHIFB3 T2PHIFB4 T2PHIFB5 T2PHIFB6 T2PHIFB7;

Missing are all (-99); IDVAR = ID;

**Analysis:** Estimator = WLSMV;

Model:

CWB BY T2CHIFB1 T2CHIFB2 T2CHIFB3 T2CHIFB4 T2CHIFB5 T2CHIFB6 T2CHIFB7;

CGWB BY T2PHIFB1 T2PHIFB2 T2PHIFB3 T2PHIFB4 T2PHIFB5 T2PHIFB6 T2PHIFB7; T2PHIFB4 with T2PHIFB5;

CGMH BY T2PSDHYP T2PSDEMO T2PSDCON T2PSDPPR; CWB with CGWB CGMH;

CGWB with CGMH;

**Output:** Sampstat; Standardized;

**Longitudinal Measurement Invariance Models**

**Child HIFAMS Questionnaire**

**Configural**

**Variable:** Names are ID T1CHIFB1 T1CHIFB2 T1CHIFB3 T1CHIFB4 T1CHIFB5 T1CHIFB6 T1CHIFB7 T2CHIFB1T2CHIFB2 T2CHIFB3 T2CHIFB4 T2CHIFB5 T2CHIFB6 T2CHIFB7;

Usevar = T1CHIFB1 T1CHIFB2 T1CHIFB3 T1CHIFB4 T1CHIFB5 T1CHIFB6 T1CHIFB7 T2CHIFB1T2CHIFB2 T2CHIFB3 T2CHIFB4 T2CHIFB5 T2CHIFB6 T2CHIFB7;

Categorical are T1CHIFB1 T1CHIFB2 T1CHIFB3 T1CHIFB4 T1CHIFB5 T1CHIFB6 T1CHIFB7 T2CHIFB1T2CHIFB2 T2CHIFB3 T2CHIFB4 T2CHIFB5 T2CHIFB6 T2CHIFB7;

Missing are all (-99); IDVAR = ID;

**Analysis:** Estimator = WLSMV;

**Model:**

!Time 1

CWBT1 BY T1CHIFB1 T1CHIFB2 T1CHIFB3 T1CHIFB4 T1CHIFB5 T1CHIFB6 T1CHIFB7;

!Time 2

CWBT2 BY T2CHIFB1 T2CHIFB2 T2CHIFB3 T2CHIFB4 T2CHIFB5 T2CHIFB6

T2CHIFB7;

!Thresholds

[T1CHIFB1$1@0]; [T1CHIFB2$1]; [T1CHIFB3$1]; [T1CHIFB4$1]; [T1CHIFB5$1]; [T1CHIFB6$1]; [T1CHIFB7$1]; [T2CHIFB1$1@0]; [T2CHIFB2$1]; [T2CHIFB3$1];

[T2CHIFB4$1]; [T2CHIFB5$1]; [T2CHIFB6$1]; [T2CHIFB7$1];

!Correlated Residuals

T1CHIFB1 with T2CHIFB1; T1CHIFB2 with T2CHIFB2; T1CHIFB3 with T2CHIFB3; T1CHIFB4 with T2CHIFB4; T1CHIFB5 with T2CHIFB5; T1CHIFB6 with T2CHIFB6; T1CHIFB7 with T2CHIFB7;

!Estimate latent factor means

[CWBT1 CWBT2];

!Estimate latent factor variances

CWBT1 CWBT2;

!Allow t1 and t2 to correlate

CWBT1 with CWBT2;

**Output:** Sampstat; Modindices (all); Standardized; Cinterval;

**Weak**

**Variable:** Names are ID T1CHIFB1 T1CHIFB2 T1CHIFB3 T1CHIFB4 T1CHIFB5 T1CHIFB6 T1CHIFB7 T2CHIFB1T2CHIFB2 T2CHIFB3 T2CHIFB4 T2CHIFB5 T2CHIFB6 T2CHIFB7;

Usevar = T1CHIFB1 T1CHIFB2 T1CHIFB3 T1CHIFB4 T1CHIFB5 T1CHIFB6 T1CHIFB7 T2CHIFB1T2CHIFB2 T2CHIFB3 T2CHIFB4 T2CHIFB5 T2CHIFB6 T2CHIFB7;

Categorical are T1CHIFB1 T1CHIFB2 T1CHIFB3 T1CHIFB4 T1CHIFB5 T1CHIFB6 T1CHIFB7 T2CHIFB1T2CHIFB2 T2CHIFB3 T2CHIFB4 T2CHIFB5 T2CHIFB6 T2CHIFB7;

Missing are all (-99); IDVAR = ID;

**Analysis:** Estimator = WLSMV;

**Model:**

!Time 1

CWBT1 BY T1CHIFB1 T1CHIFB2 (1) T1CHIFB3 (2) T1CHIFB4 (3) T1CHIFB5 (4) T1CHIFB6 (5) T1CHIFB7 (6);

!Time 2

CWBT2 BY T2CHIFB1 T2CHIFB2 (1) T2CHIFB3 (2) T2CHIFB4 (3) T2CHIFB5 (4) T2CHIFB6 (5) T2CHIFB7 (6);

!Thresholds

[T1CHIFB1$1@0]; [T1CHIFB2$1]; [T1CHIFB3$1]; [T1CHIFB4$1]; [T1CHIFB5$1]; [T1CHIFB6$1]; [T1CHIFB7$1]; [T2CHIFB1$1@0]; [T2CHIFB2$1]; [T2CHIFB3$1];

[T2CHIFB4$1]; [T2CHIFB5$1]; [T2CHIFB6$1]; [T2CHIFB7$1];

!Correlated Residuals

T1CHIFB1 with T2CHIFB1; T1CHIFB2 with T2CHIFB2; T1CHIFB3 with T2CHIFB3; T1CHIFB4 with T2CHIFB4; T1CHIFB5 with T2CHIFB5; T1CHIFB6 with T2CHIFB6; T1CHIFB7 with T2CHIFB7;

!Estimate latent factor means

[CWBT1 CWBT2];

!Estimate latent factor variances

CWBT1 CWBT2;

!Allow t1 and t2 to correlate

CWBT1 with CWBT2;

**Output:** Sampstat; Modindices (all); Standardized; Cinterval;

**Strong**

**Variable:** Names are ID T1CHIFB1 T1CHIFB2 T1CHIFB3 T1CHIFB4 T1CHIFB5 T1CHIFB6 T1CHIFB7 T2CHIFB1T2CHIFB2 T2CHIFB3 T2CHIFB4 T2CHIFB5 T2CHIFB6 T2CHIFB7;

Usevar = T1CHIFB1 T1CHIFB2 T1CHIFB3 T1CHIFB4 T1CHIFB5 T1CHIFB6 T1CHIFB7 T2CHIFB1T2CHIFB2 T2CHIFB3 T2CHIFB4 T2CHIFB5 T2CHIFB6 T2CHIFB7;

Categorical are T1CHIFB1 T1CHIFB2 T1CHIFB3 T1CHIFB4 T1CHIFB5 T1CHIFB6 T1CHIFB7 T2CHIFB1T2CHIFB2 T2CHIFB3 T2CHIFB4 T2CHIFB5 T2CHIFB6 T2CHIFB7;

Missing are all (-99); IDVAR = ID;

**Analysis:** Estimator = WLSMV;

**Model:**

!Time 1

CWBT1 BY T1CHIFB1 T1CHIFB2 (1) T1CHIFB3 (2) T1CHIFB4 (3) T1CHIFB5 (4) T1CHIFB6 (5) T1CHIFB7 (6);

!Time 2

CWBT2 BY T2CHIFB1 T2CHIFB2 (1) T2CHIFB3 (2) T2CHIFB4 (3) T2CHIFB5 (4) T2CHIFB6 (5) T2CHIFB7 (6);

!Thresholds

[T1CHIFB1$1@0]; [T1CHIFB2$1] (7); [T1CHIFB3$1] (8); [T1CHIFB4$1] (9); [T1CHIFB5$1] (10); [T1CHIFB6$1] (11); [T1CHIFB7$1] (12); [T2CHIFB1$1@0]; [T2CHIFB2$1] (7); [T2CHIFB3$1] (8); [T2CHIFB4$1] (9); [T2CHIFB5$1] (10); [T2CHIFB6$1] (11); [T2CHIFB7$1] (12);

!Correlated Residuals

T1CHIFB1 with T2CHIFB1; T1CHIFB2 with T2CHIFB2; T1CHIFB3 with T2CHIFB3; T1CHIFB4 with T2CHIFB4; T1CHIFB5 with T2CHIFB5; T1CHIFB6 with T2CHIFB6; T1CHIFB7 with T2CHIFB7;

!Estimate latent factor means

[CWBT1 CWBT2];

!Estimate latent factor variances

CWBT1 CWBT2;

!Allow t1 and t2 to correlate

CWBT1 with CWBT2;

**Output:** Sampstat; Modindices (all); Standardized; Cinterval;

**Partial**

**Variable:** Names are ID T1CHIFB1 T1CHIFB2 T1CHIFB3 T1CHIFB4 T1CHIFB5 T1CHIFB6 T1CHIFB7 T2CHIFB1T2CHIFB2 T2CHIFB3 T2CHIFB4 T2CHIFB5 T2CHIFB6 T2CHIFB7;

Usevar = T1CHIFB1 T1CHIFB2 T1CHIFB3 T1CHIFB4 T1CHIFB5 T1CHIFB6 T1CHIFB7 T2CHIFB1T2CHIFB2 T2CHIFB3 T2CHIFB4 T2CHIFB5 T2CHIFB6 T2CHIFB7;

Categorical are T1CHIFB1 T1CHIFB2 T1CHIFB3 T1CHIFB4 T1CHIFB5 T1CHIFB6 T1CHIFB7 T2CHIFB1T2CHIFB2 T2CHIFB3 T2CHIFB4 T2CHIFB5 T2CHIFB6 T2CHIFB7;

Missing are all (-99); IDVAR = ID;

**Analysis:** Estimator = WLSMV;

**Model:**

!Time 1

CWBT1 BY T1CHIFB1 T1CHIFB2 (1) T1CHIFB3 (2) T1CHIFB4 (3) T1CHIFB5 (4) T1CHIFB6 (5) T1CHIFB7 (6);

!Time 2

CWBT2 BY T2CHIFB1 T2CHIFB2 (1) T2CHIFB3 (2) T2CHIFB4 (3) T2CHIFB5 (4) T2CHIFB6 (5) T2CHIFB7 (6);

!Thresholds

[T1CHIFB1$1@0]; [T1CHIFB2$1] ; [T1CHIFB3$1] (8); [T1CHIFB4$1] (9); [T1CHIFB5$1] (10); [T1CHIFB6$1] ; [T1CHIFB7$1] (12); [T2CHIFB1$1@0]; [T2CHIFB2$1] ; [T2CHIFB3$1] (8); [T2CHIFB4$1] (9); [T2CHIFB5$1] (10); [T2CHIFB6$1] ; [T2CHIFB7$1] (12);

!Correlated Residuals

T1CHIFB1 with T2CHIFB1; T1CHIFB2 with T2CHIFB2; T1CHIFB3 with T2CHIFB3; T1CHIFB4 with T2CHIFB4; T1CHIFB5 with T2CHIFB5; T1CHIFB6 with T2CHIFB6; T1CHIFB7 with T2CHIFB7;

!Estimate latent factor means

[CWBT1 CWBT2];

!Estimate latent factor variances

CWBT1 CWBT2;

!Allow t1 and t2 to correlate

CWBT1 with CWBT2;

**Output:** Sampstat; Modindices (all); Standardized; Cinterval;

**Caregiver HIFAMS Questionnaire**

**Configural**

**Variable:** Names are ID T1PHIFB1 T1PHIFB2 T1PHIFB3 T1PHIFB4 T1PHIFB5 T1PHIFB6 T1PHIFB7 T2PHIFB1T2PHIFB2 T2PHIFB3 T2PHIFB4 T2PHIFB5 T2PHIFB6 T2PHIFB7;

Usevar are T1PHIFB1 T1PHIFB2 T1PHIFB3 T1PHIFB4 T1PHIFB5 T1PHIFB6 T1PHIFB7 T2PHIFB1T2PHIFB2 T2PHIFB3 T2PHIFB4 T2PHIFB5 T2PHIFB6 T2PHIFB7;

Categorical are T1PHIFB1 T1PHIFB2 T1PHIFB3 T1PHIFB4 T1PHIFB5 T1PHIFB6 T1PHIFB7 T2PHIFB1T2PHIFB2 T2PHIFB3 T2PHIFB4 T2PHIFB5 T2PHIFB6 T2PHIFB7;

Missing are all (-99); IDVAR = ID;

**Analysis:** Estimator = WLSMV;

**Model:**

!Time 1

PCWBT1 BY T1PHIFB1 T1PHIFB2 T1PHIFB3 T1PHIFB4 T1PHIFB5 T1PHIFB6 T1PHIFB7; T1PHIFB4 with T1PHIFB5;

!Time 2

PCWBT2 BY T2PHIFB1T2PHIFB2 T2PHIFB3 T2PHIFB4 T2PHIFB5 T2PHIFB6 T2PHIFB7; T2PHIFB4 with T2PHIFB5;

!Thresholds

[T1PHIFB1$1@0]; [T1PHIFB2$1]; [T1PHIFB3$1]; [T1PHIFB4$1]; [T1PHIFB5$1] ; [T1PHIFB6$1] ; [T1PHIFB7$1]; [T2PHIFB1$1@0]; [T2PHIFB2$1] ; [T2PHIFB3$1]; [T2PHIFB4$1]; [T T2PHIFB5$1] ; [T2PHIFB6$1] ; [T2PHIFB7$1] ;

!Correlated Residuals

T1PHIFB1 with T2PHIFB1; T1PHIFB2 with T2PHIFB2; T1PHIFB3 with T2PHIFB3; T1PHIFB4 with T2PHIFB4; T1PHIFB5 with T2PHIFB5; T1PHIFB6 with T2PHIFB6; T1PHIFB7 with T2PHIFB7;

!Estimate latent factor means

[PCWBT1]; [PCWBT2];

!Estimate latent factor variances

PCWBT1; PCWBT2;

!Allow t1 and t2 to correlate

PCWBT1 with PCWBT2;

**Output:** Sampstat; Modindices (all); Standardized; Cinterval;

**Weak**

**Variable:** Names are ID T1PHIFB1 T1PHIFB2 T1PHIFB3 T1PHIFB4 T1PHIFB5 T1PHIFB6 T1PHIFB7 T2PHIFB1T2PHIFB2 T2PHIFB3 T2PHIFB4 T2PHIFB5 T2PHIFB6 T2PHIFB7;

Usevar are T1PHIFB1 T1PHIFB2 T1PHIFB3 T1PHIFB4 T1PHIFB5 T1PHIFB6 T1PHIFB7 T2PHIFB1T2PHIFB2 T2PHIFB3 T2PHIFB4 T2PHIFB5 T2PHIFB6 T2PHIFB7;

Categorical are T1PHIFB1 T1PHIFB2 T1PHIFB3 T1PHIFB4 T1PHIFB5 T1PHIFB6 T1PHIFB7 T2PHIFB1T2PHIFB2 T2PHIFB3 T2PHIFB4 T2PHIFB5 T2PHIFB6 T2PHIFB7;

Missing are all (-99); IDVAR = ID;

**Analysis:** Estimator = WLSMV;

**Model:**

!Time 1

PCWBT1 BY T1PHIFB1 T1PHIFB2 (1) T1PHIFB3 (2) T1PHIFB4 (3) T1PHIFB5 (4) T1PHIFB6 (5) T1PHIFB7 (6); T1PHIFB4 with T1PHIFB5;

!Time 2

PCWBT2 BY T2PHIFB1T2PHIFB2 (1) T2PHIFB3 (2) T2PHIFB4 (3) T2PHIFB5 (4) T2PHIFB6 (5) T2PHIFB7 (6); T2PHIFB4 with T2PHIFB5;

!Thresholds

[T1PHIFB1$1@0]; [T1PHIFB2$1]; [T1PHIFB3$1]; [T1PHIFB4$1]; [T1PHIFB5$1] ; [T1PHIFB6$1] ; [T1PHIFB7$1]; [T2PHIFB1$1@0]; [T2PHIFB2$1] ; [T2PHIFB3$1]; [T2PHIFB4$1]; [T T2PHIFB5$1] ; [T2PHIFB6$1] ; [T2PHIFB7$1] ;

!Correlated Residuals

T1PHIFB1 with T2PHIFB1; T1PHIFB2 with T2PHIFB2; T1PHIFB3 with T2PHIFB3; T1PHIFB4 with T2PHIFB4; T1PHIFB5 with T2PHIFB5; T1PHIFB6 with T2PHIFB6; T1PHIFB7 with T2PHIFB7;

!Estimate latent factor means

[PCWBT1]; [PCWBT2];

!Estimate latent factor variances

PCWBT1; PCWBT2;

!Allow t1 and t2 to correlate

PCWBT1 with PCWBT2;

**Output:** Sampstat; Modindices (all); Standardized; Cinterval;

**Strong**

**Variable:** Names are ID T1PHIFB1 T1PHIFB2 T1PHIFB3 T1PHIFB4 T1PHIFB5 T1PHIFB6 T1PHIFB7 T2PHIFB1T2PHIFB2 T2PHIFB3 T2PHIFB4 T2PHIFB5 T2PHIFB6 T2PHIFB7;

Usevar are T1PHIFB1 T1PHIFB2 T1PHIFB3 T1PHIFB4 T1PHIFB5 T1PHIFB6 T1PHIFB7 T2PHIFB1T2PHIFB2 T2PHIFB3 T2PHIFB4 T2PHIFB5 T2PHIFB6 T2PHIFB7;

Categorical are T1PHIFB1 T1PHIFB2 T1PHIFB3 T1PHIFB4 T1PHIFB5 T1PHIFB6 T1PHIFB7 T2PHIFB1T2PHIFB2 T2PHIFB3 T2PHIFB4 T2PHIFB5 T2PHIFB6 T2PHIFB7;

Missing are all (-99); IDVAR = ID;

**Analysis:** Estimator = WLSMV;

**Model:**

!Time 1

PCWBT1 BY T1PHIFB1 T1PHIFB2 (1) T1PHIFB3 (2) T1PHIFB4 (3) T1PHIFB5 (4) T1PHIFB6 (5) T1PHIFB7 (6); T1PHIFB4 with T1PHIFB5;

!Time 2

PCWBT2 BY T2PHIFB1T2PHIFB2 (1) T2PHIFB3 (2) T2PHIFB4 (3) T2PHIFB5 (4) T2PHIFB6 (5) T2PHIFB7 (6); T2PHIFB4 with T2PHIFB5;

!Thresholds

[T1PHIFB1$1@0]; [T1PHIFB2$1] (7); [T1PHIFB3$1] (8); [T1PHIFB4$1] (9); [T1PHIFB5$1] (10); [T1PHIFB6$1] (11); [T1PHIFB7$1] (12); [T2PHIFB1$1@0]; [T2PHIFB2$1] (7); [T2PHIFB3$1] (8); [T2PHIFB4$1] (9); [T T2PHIFB5$1] (10) ; [T2PHIFB6$1] (11); [T2PHIFB7$1] (12) ;

!Correlated Residuals

T1PHIFB1 with T2PHIFB1; T1PHIFB2 with T2PHIFB2; T1PHIFB3 with T2PHIFB3; T1PHIFB4 with T2PHIFB4; T1PHIFB5 with T2PHIFB5; T1PHIFB6 with T2PHIFB6; T1PHIFB7 with T2PHIFB7;

!Estimate latent factor means

[PCWBT1]; [PCWBT2];

!Estimate latent factor variances

PCWBT1; PCWBT2;

!Allow t1 and t2 to correlate

PCWBT1 with PCWBT2;

**Output:** Sampstat; Modindices (all); Standardized; Cinterval;

**Covariance**

**Variable:** Names are ID T1PHIFB1 T1PHIFB2 T1PHIFB3 T1PHIFB4 T1PHIFB5 T1PHIFB6 T1PHIFB7 T2PHIFB1T2PHIFB2 T2PHIFB3 T2PHIFB4 T2PHIFB5 T2PHIFB6 T2PHIFB7;

Usevar are T1PHIFB1 T1PHIFB2 T1PHIFB3 T1PHIFB4 T1PHIFB5 T1PHIFB6 T1PHIFB7 T2PHIFB1T2PHIFB2 T2PHIFB3 T2PHIFB4 T2PHIFB5 T2PHIFB6 T2PHIFB7;

Categorical are T1PHIFB1 T1PHIFB2 T1PHIFB3 T1PHIFB4 T1PHIFB5 T1PHIFB6 T1PHIFB7 T2PHIFB1T2PHIFB2 T2PHIFB3 T2PHIFB4 T2PHIFB5 T2PHIFB6 T2PHIFB7;

Missing are all (-99); IDVAR = ID;

**Analysis:** Estimator = WLSMV;

**Model:**

!Time 1

PCWBT1 BY T1PHIFB1 T1PHIFB2 (1) T1PHIFB3 (2) T1PHIFB4 (3) T1PHIFB5 (4) T1PHIFB6 (5) T1PHIFB7 (6); T1PHIFB4 with T1PHIFB5 (13);

!Time 2

PCWBT2 BY T2PHIFB1T2PHIFB2 (1) T2PHIFB3 (2) T2PHIFB4 (3) T2PHIFB5 (4) T2PHIFB6 (5) T2PHIFB7 (6); T2PHIFB4 with T2PHIFB5 (13);

!Thresholds

[T1PHIFB1$1@0]; [T1PHIFB2$1] (7); [T1PHIFB3$1] (8); [T1PHIFB4$1] (9); [T1PHIFB5$1] (10); [T1PHIFB6$1] (11); [T1PHIFB7$1] (12); [T2PHIFB1$1@0]; [T2PHIFB2$1] (7); [T2PHIFB3$1] (8); [T2PHIFB4$1] (9); [T T2PHIFB5$1] (10) ; [T2PHIFB6$1] (11); [T2PHIFB7$1] (12) ;

!Correlated Residuals

T1PHIFB1 with T2PHIFB1; T1PHIFB2 with T2PHIFB2; T1PHIFB3 with T2PHIFB3; T1PHIFB4 with T2PHIFB4; T1PHIFB5 with T2PHIFB5; T1PHIFB6 with T2PHIFB6; T1PHIFB7 with T2PHIFB7;

!Estimate latent factor means

[PCWBT1]; [PCWBT2];

!Estimate latent factor variances

PCWBT1; PCWBT2;

!Allow t1 and t2 to correlate

PCWBT1 with PCWBT2;

**Output:** Sampstat; Modindices (all); Standardized; Cinterval;

**Caregiver-Rated Child SDQ**

**Configural**

**Variable:** Names are ID T1PSDHYP T1PSDEMO T1PSDCON T1PSDPPR T2PSDHYP T2PSDEMO T2PSDCON T2PSDPPR;

Usevar are T1PSDHYP T1PSDEMO T1PSDCON T1PSDPPR T2PSDHYP T2PSDEMO T2PSDCON T2PSDPPR;

Missing are all (-99); IDVAR = ID;

**Analysis:** Estimator = MLR;

**Model:**

!Time 1

CMHT1 BY T1PSDHYP T1PSDEMO T1PSDCON T1PSDPPR;

!Time 2

CMHT2 BY T2PSDHYP T2PSDEMO T2PSDCON T2PSDPPR;

!Intercepts

[T1PSDHYP@0]; [T1PSDEMO]; [T1PSDCON]; [T1PSDPPR];

[T2PSDHYP@0]; [T2PSDEMO]; [T2PSDCON]; [T2PSDPPR];

!Residuals

T1PSDHYP; T1PSDEMO; T1PSDCON; T1PSDPPR;

T2PSDHYP; T2PSDEMO; T2PSDCON; T2PSDPPR;

!Correlated Residuals

T1PSDHYP with T2PSDHYP;

T1PSDEMO with T2PSDEMO;

T1PSDCON with T2PSDCON;

T1PSDPPR with T2PSDPPR;

!Estimate latent factor means

[CMHT1]; [CMHT2];

!Estimate latent factor variances

CMHT1; CMHT2;

**Output:** Sampstat; Modindices (all); Standardized; Cinterval;

**Weak**

**Variable:** Names are ID T1PSDHYP T1PSDEMO T1PSDCON T1PSDPPR T2PSDHYP T2PSDEMO T2PSDCON T2PSDPPR;

Usevar are T1PSDHYP T1PSDEMO T1PSDCON T1PSDPPR T2PSDHYP T2PSDEMO T2PSDCON T2PSDPPR;

Missing are all (-99); IDVAR = ID;

**Analysis:** Estimator = MLR;

**Model:**

!Time 1

CMHT1 BY T1PSDHYP T1PSDEMO (1) T1PSDCON (2) T1PSDPPR (3);

!Time 2

CMHT2 BY T2PSDHYP T2PSDEMO (1) T2PSDCON (2) T2PSDPPR (3);

!Intercepts

[T1PSDHYP@0]; [T1PSDEMO]; [T1PSDCON]; [T1PSDPPR];

[T2PSDHYP@0]; [T2PSDEMO]; [T2PSDCON]; [T2PSDPPR];

!Residuals

T1PSDHYP; T1PSDEMO; T1PSDCON; T1PSDPPR;

T2PSDHYP; T2PSDEMO; T2PSDCON; T2PSDPPR;

!Correlated Residuals

T1PSDHYP with T2PSDHYP;

T1PSDEMO with T2PSDEMO;

T1PSDCON with T2PSDCON;

T1PSDPPR with T2PSDPPR;

!Estimate latent factor means

[CMHT1]; [CMHT2];

!Estimate latent factor variances

CMHT1; CMHT2;

**Output:** Sampstat; Modindices (all); Standardized; Cinterval;

**Strong**

**Variable:** Names are ID T1PSDHYP T1PSDEMO T1PSDCON T1PSDPPR T2PSDHYP T2PSDEMO T2PSDCON T2PSDPPR;

Usevar are T1PSDHYP T1PSDEMO T1PSDCON T1PSDPPR T2PSDHYP T2PSDEMO T2PSDCON T2PSDPPR;

Missing are all (-99); IDVAR = ID;

**Analysis:** Estimator = MLR;

**Model:**

!Time 1

CMHT1 BY T1PSDHYP T1PSDEMO (1) T1PSDCON (2) T1PSDPPR (3);

!Time 2

CMHT2 BY T2PSDHYP T2PSDEMO (1) T2PSDCON (2) T2PSDPPR (3);

!Intercepts

[T1PSDHYP@0]; [T1PSDEMO](4); [T1PSDCON](5); [T1PSDPPR](6);

[T2PSDHYP@0]; [T2PSDEMO](4); [T2PSDCON](5); [T2PSDPPR](6);

!Residuals

T1PSDHYP; T1PSDEMO; T1PSDCON; T1PSDPPR;

T2PSDHYP; T2PSDEMO; T2PSDCON; T2PSDPPR;

!Correlated Residuals

T1PSDHYP with T2PSDHYP;

T1PSDEMO with T2PSDEMO;

T1PSDCON with T2PSDCON;

T1PSDPPR with T2PSDPPR;

!Estimate latent factor means

[CMHT1]; [CMHT2];

!Estimate latent factor variances

CMHT1; CMHT2;

**Output:** Sampstat; Modindices (all); Standardized; Cinterval;

**Residuals**

**Variable:** Names are ID T1PSDHYP T1PSDEMO T1PSDCON T1PSDPPR T2PSDHYP T2PSDEMO T2PSDCON T2PSDPPR;

Usevar are T1PSDHYP T1PSDEMO T1PSDCON T1PSDPPR T2PSDHYP T2PSDEMO T2PSDCON T2PSDPPR;

Missing are all (-99); IDVAR = ID;

**Analysis:** Estimator = MLR;

**Model:**

!Time 1

CMHT1 BY T1PSDHYP T1PSDEMO (1) T1PSDCON (2) T1PSDPPR (3);

!Time 2

CMHT2 BY T2PSDHYP T2PSDEMO (1) T2PSDCON (2) T2PSDPPR (3);

!Intercepts

[T1PSDHYP@0]; [T1PSDEMO](4); [T1PSDCON](5); [T1PSDPPR](6);

[T2PSDHYP@0]; [T2PSDEMO](4); [T2PSDCON](5); [T2PSDPPR](6);

!Residuals

T1PSDHYP (7); T1PSDEMO (8); T1PSDCON (9); T1PSDPPR (10);

T2PSDHYP (7); T2PSDEMO (8); T2PSDCON (9); T2PSDPPR (10);

!Correlated Residuals

T1PSDHYP with T2PSDHYP;

T1PSDEMO with T2PSDEMO;

T1PSDCON with T2PSDCON;

T1PSDPPR with T2PSDPPR;

!Estimate latent factor means

[CMHT1]; [CMHT2];

!Estimate latent factor variances

CMHT1; CMHT2;

**Output:** Sampstat; Modindices (all); Standardized; Cinterval;

**Autoregressive Model (Model 9)**

**Data:**

File = RoNWBlist.dat; Type = Imputation;

**Variable:** Names are T2CGEND T1CAGEY T1CHIFB1 T1CHIFB2 T1CHIFB3 T1CHIFB4 T1CHIFB5 T1CHIFB6 T1CHIFB7 T2CHIFB1 T2CHIFB2 T2CHIFB3 T2CHIFB4 T2CHIFB5 T2CHIFB6 T2CHIFB7 T1PHIFB1 T1PHIFB2 T1PHIFB3 T1PHIFB4 T1PHIFB5 T1PHIFB6 T1PHIFB7 T2PHIFB1 T2PHIFB2 T2PHIFB3 T2PHIFB4 T2PHIFB5 T2PHIFB6 T2PHIFB7 T2THIFB1 T2THIFB2 T2THIFB3 T2THIFB4 T1PSDPRO T1PSDHYP T1PSDEMO T1PSDCON T1PSDPPR T2PSDHYP T2PSDEMO T2PSDCON T2PSDPPR T1VAZ SESZ T1EFFS T2ACADFS T2SOCFS ID;

Usevar are T1PSDHYP T1PSDEMO T1PSDCON T1PSDPPR T2PSDHYP T2PSDEMO T2PSDCON T2PSDPPR T1PHIFB1 T1PHIFB2 T1PHIFB3 T1PHIFB4 T1PHIFB5 T1PHIFB6 T1PHIFB7 T2PHIFB1T2PHIFB2 T2PHIFB3 T2PHIFB4 T2PHIFB5 T2PHIFB6 T2PHIFB7 T1CHIFB1 T1CHIFB2 T1CHIFB3 T1CHIFB4 T1CHIFB5 T1CHIFB6 T1CHIFB7 T2CHIFB1T2CHIFB2 T2CHIFB3 T2CHIFB4 T2CHIFB5 T2CHIFB6 T2CHIFB7;

Categorical are T1PHIFB1 T1PHIFB2 T1PHIFB3 T1PHIFB4 T1PHIFB5 T1PHIFB6 T1PHIFB7 T2PHIFB1T2PHIFB2 T2PHIFB3 T2PHIFB4 T2PHIFB5 T2PHIFB6 T2PHIFB7 T1CHIFB1 T1CHIFB2 T1CHIFB3 T1CHIFB4 T1CHIFB5 T1CHIFB6 T1CHIFB7 T2CHIFB1T2CHIFB2 T2CHIFB3 T2CHIFB4 T2CHIFB5 T2CHIFB6 T2CHIFB7;

Missing are all (-99); IDVAR = ID;

**Analysis:** Estimator = WLSMV;

**Model:**

!Caregiver-Rated Child SDQ

!Time 1

CMHT1 BY T1PSDHYP T1PSDEMO (1) T1PSDCON (2) T1PSDPPR (3);

!Time 2

CMHT2 BY T2PSDHYP T2PSDEMO (1) T2PSDCON (2) T2PSDPPR (3);

!Intercepts

[T1PSDHYP@0]; [T1PSDEMO](4); [T1PSDCON](5); [T1PSDPPR](6);

[T2PSDHYP@0]; [T2PSDEMO](4); [T2PSDCON](5); [T2PSDPPR](6);

!Residuals

T1PSDHYP (7); T1PSDEMO (8); T1PSDCON (9); T1PSDPPR (10);

T2PSDHYP (7); T2PSDEMO (8); T2PSDCON (9); T2PSDPPR (10);

!Correlated Residuals

T1PSDHYP with T2PSDHYP;

T1PSDEMO with T2PSDEMO;

T1PSDCON with T2PSDCON;

T1PSDPPR with T2PSDPPR;

!Estimate latent factor means and variances

[CMHT1]; [CMHT2]; CMHT1; CMHT2

!Caregiver-Rated HIFAMS

!Time 1

PCWBT1 BY T1PHIFB1 T1PHIFB2 (a) T1PHIFB3 (b) T1PHIFB4 (c) T1PHIFB5 (d) T1PHIFB6 (e) T1PHIFB7 (f); T1PHIFB4 with T1PHIFB5 (g);

!Time 2

PCWBT2 BY T2PHIFB1T2PHIFB2 (a) T2PHIFB3 (b) T2PHIFB4 (c) T2PHIFB5 (d) T2PHIFB6 (e) T2PHIFB7 (f); T2PHIFB4 with T2PHIFB5 (g);

!Thresholds

[T1PHIFB1$1@0]; [T1PHIFB2$1] (h); [T1PHIFB3$1] (i); [T1PHIFB4$1] (j); [T1PHIFB5$1] (k); [T1PHIFB6$1] (l); [T1PHIFB7$1] (m); [T2PHIFB1$1@0]; [T2PHIFB2$1] (h); [T2PHIFB3$1] (i); [T2PHIFB4$1] (j); [T T2PHIFB5$1] (k) ; [T2PHIFB6$1] (l); [T2PHIFB7$1] (m) ;

!Correlated Residuals

T1PHIFB1 with T2PHIFB1; T1PHIFB2 with T2PHIFB2; T1PHIFB3 with T2PHIFB3; T1PHIFB4 with T2PHIFB4; T1PHIFB5 with T2PHIFB5; T1PHIFB6 with T2PHIFB6; T1PHIFB7 with T2PHIFB7;

!Estimate latent factor means and variances

[PCWBT1]; [PCWBT2]; PCWBT1; PCWBT2;

!Child-Rated HIFAMS

!Time 1

CWBT1 BY T1CHIFB1 T1CHIFB2 (p1) T1CHIFB3 (p2) T1CHIFB4 (p3) T1CHIFB5 (p4) T1CHIFB6 (p5) T1CHIFB7 (p6);

!Time 2

CWBT2 BY T2CHIFB1 T2CHIFB2 (p1) T2CHIFB3 (p2) T2CHIFB4 (p3) T2CHIFB5 (p4) T2CHIFB6 (p5) T2CHIFB7 (p6);

!Thresholds

[T1CHIFB1$1@0]; [T1CHIFB2$1] ; [T1CHIFB3$1] (p8); [T1CHIFB4$1] (p9); [T1CHIFB5$1] (p10); [T1CHIFB6$1] ; [T1CHIFB7$1] (p12); [T2CHIFB1$1@0]; [T2CHIFB2$1] ; [T2CHIFB3$1] (p8); [T2CHIFB4$1] (p9); [T2CHIFB5$1] (p10); [T2CHIFB6$1] ; [T2CHIFB7$1] (p12);

!Correlated Residuals

T1CHIFB1 with T2CHIFB1; T1CHIFB2 with T2CHIFB2; T1CHIFB3 with T2CHIFB3; T1CHIFB4 with T2CHIFB4; T1CHIFB5 with T2CHIFB5; T1CHIFB6 with T2CHIFB6; T1CHIFB7 with T2CHIFB7;

!Estimate latent factor means and variances

[CWBT1 CWBT2]; CWBT1; CWBT2;

!Within-time covariances

CWBT1 with PCWBT1 CMHT1; PCWBT1 with CMHT1;

CWBT2 with PCWBT2 CMHT2; PCWBT2 with CMHT2;

!Autoregressive Paths

CWBT2 on CWBT1; PCWBT2 on PCWBT1; CMHT2 on CMHT1;

**Output:** Sampstat; Standardized; Cinterval;

**Univariate Latent Change Score Models**

**Model 10: Child-Rated HIFAMS**

**Data:** File = RoNWBlist.dat; Type = Imputation;

**Variable:** Names are T2CGEND T1CAGEY T1CHIFB1 T1CHIFB2 T1CHIFB3 T1CHIFB4 T1CHIFB5 T1CHIFB6 T1CHIFB7 T2CHIFB1 T2CHIFB2 T2CHIFB3 T2CHIFB4 T2CHIFB5 T2CHIFB6 T2CHIFB7 T1PHIFB1 T1PHIFB2 T1PHIFB3 T1PHIFB4 T1PHIFB5 T1PHIFB6 T1PHIFB7 T2PHIFB1 T2PHIFB2 T2PHIFB3 T2PHIFB4 T2PHIFB5 T2PHIFB6 T2PHIFB7 T2THIFB1 T2THIFB2 T2THIFB3 T2THIFB4 T1PSDPRO T1PSDHYP T1PSDEMO T1PSDCON T1PSDPPR T2PSDHYP T2PSDEMO T2PSDCON T2PSDPPR T1VAZ SESZ T1EFFS T2ACADFS T2SOCFS ID;

Usevar = T1CHIFB1 T1CHIFB2 T1CHIFB3 T1CHIFB4 T1CHIFB5 T1CHIFB6 T1CHIFB7 T2CHIFB1T2CHIFB2 T2CHIFB3 T2CHIFB4 T2CHIFB5 T2CHIFB6 T2CHIFB7;

Categorical are T1CHIFB1 T1CHIFB2 T1CHIFB3 T1CHIFB4 T1CHIFB5 T1CHIFB6 T1CHIFB7 T2CHIFB1T2CHIFB2 T2CHIFB3 T2CHIFB4 T2CHIFB5 T2CHIFB6 T2CHIFB7;

Missing are all (-99); IDVAR = ID;

**Analysis:** Estimator = WLSMV;

**Model:**

!Time 1

CWBT1 BY T1CHIFB1 T1CHIFB2 (1) T1CHIFB3 (2) T1CHIFB4 (3) T1CHIFB5 (4) T1CHIFB6 (5) T1CHIFB7 (6);

!Time 2

CWBT2 BY T2CHIFB1 T2CHIFB2 (1) T2CHIFB3 (2) T2CHIFB4 (3) T2CHIFB5 (4) T2CHIFB6 (5) T2CHIFB7 (6);

!Thresholds

[T1CHIFB1$1@0]; [T1CHIFB2$1] ; [T1CHIFB3$1] (8); [T1CHIFB4$1] (9); [T1CHIFB5$1] (10); [T1CHIFB6$1] ; [T1CHIFB7$1] (12); [T2CHIFB1$1@0]; [T2CHIFB2$1] ; [T2CHIFB3$1] (8); [T2CHIFB4$1] (9); [T2CHIFB5$1] (10); [T2CHIFB6$1] ; [T2CHIFB7$1] (12);

!Correlated Residuals

T1CHIFB1 with T2CHIFB1; T1CHIFB2 with T2CHIFB2; T1CHIFB3 with T2CHIFB3; T1CHIFB4 with T2CHIFB4; T1CHIFB5 with T2CHIFB5; T1CHIFB6 with T2CHIFB6; T1CHIFB7 with T2CHIFB7;

CWBT2 on CWBT1@1; !Fixed regression of T2 on T1

diffCWB BY CWBT2@1; !Latent Change Factor for Child Wellbeing

[CWBT2@0]; !Fixed latent mean of T2 to 0

CWBT2@0; !Fixed latent variance of T2 to 0

[diffCWB]; !Estimate mean of latent change factor

[CWBT1]; !Estimate mean of T1 latent factor

diffCWB; !Estimate variance of latent change factor

CWBT1; !Estimate variance of T1 latent factor

diffCWB with CWBT1; !Allow T1 factor to correlate with latent change factor

**Output:** Sampstat; Standardized; Cinterval;

**Model 11: Caregiver-Rated HIFAMS**

**Data:** File = RoNWBlist.dat; Type = Imputation;

**Variable:** Names are T2CGEND T1CAGEY T1CHIFB1 T1CHIFB2 T1CHIFB3 T1CHIFB4 T1CHIFB5 T1CHIFB6 T1CHIFB7 T2CHIFB1 T2CHIFB2 T2CHIFB3 T2CHIFB4 T2CHIFB5 T2CHIFB6 T2CHIFB7 T1PHIFB1 T1PHIFB2 T1PHIFB3 T1PHIFB4 T1PHIFB5 T1PHIFB6 T1PHIFB7 T2PHIFB1 T2PHIFB2 T2PHIFB3 T2PHIFB4 T2PHIFB5 T2PHIFB6 T2PHIFB7 T2THIFB1 T2THIFB2 T2THIFB3 T2THIFB4 T1PSDPRO T1PSDHYP T1PSDEMO T1PSDCON T1PSDPPR T2PSDHYP T2PSDEMO T2PSDCON T2PSDPPR T1VAZ SESZ T1EFFS T2ACADFS T2SOCFS ID;

Usevar are T1PHIFB1 T1PHIFB2 T1PHIFB3 T1PHIFB4 T1PHIFB5 T1PHIFB6 T1PHIFB7 T2PHIFB1T2PHIFB2 T2PHIFB3 T2PHIFB4 T2PHIFB5 T2PHIFB6 T2PHIFB7;

Categorical are T1PHIFB1 T1PHIFB2 T1PHIFB3 T1PHIFB4 T1PHIFB5 T1PHIFB6 T1PHIFB7 T2PHIFB1T2PHIFB2 T2PHIFB3 T2PHIFB4 T2PHIFB5 T2PHIFB6 T2PHIFB7;

Missing are all (-99); IDVAR = ID;

**Analysis:** Estimator = WLSMV;

**Model:**

!Time 1

PCWBT1 BY T1PHIFB1 T1PHIFB2 (a) T1PHIFB3 (b) T1PHIFB4 (c) T1PHIFB5 (d) T1PHIFB6 (e) T1PHIFB7 (f); T1PHIFB4 with T1PHIFB5 (g);

!Time 2

PCWBT2 BY T2PHIFB1T2PHIFB2 (a) T2PHIFB3 (b) T2PHIFB4 (c) T2PHIFB5 (d) T2PHIFB6 (e) T2PHIFB7 (f); T2PHIFB4 with T2PHIFB5 (g);

!Thresholds

[T1PHIFB1$1@0]; [T1PHIFB2$1] (h); [T1PHIFB3$1] (i); [T1PHIFB4$1] (j); [T1PHIFB5$1] (k); [T1PHIFB6$1] (l); [T1PHIFB7$1] (m); [T2PHIFB1$1@0]; [T2PHIFB2$1] (h); [T2PHIFB3$1] (i); [T2PHIFB4$1] (j); [T T2PHIFB5$1] (k) ; [T2PHIFB6$1] (l); [T2PHIFB7$1] (m) ;

!Correlated Residuals

T1PHIFB1 with T2PHIFB1; T1PHIFB2 with T2PHIFB2; T1PHIFB3 with T2PHIFB3; T1PHIFB4 with T2PHIFB4; T1PHIFB5 with T2PHIFB5; T1PHIFB6 with T2PHIFB6; T1PHIFB7 with T2PHIFB7;

PCWBT2 on PCWBT1@1; !Fixed regression of T2 on T1

diffPCWB BY PCWBT2@1; !Latent Change Factor for Caregiver-Rated Child Wellbeing

[PCWBT2@0]; !Fixed latent mean of T2 to 0

PCWBT2@0; !Fixed latent variance of T2 to 0

[diffPCWB]; !Estimate mean of latent change factor

[PCWBT1]; !Estimate mean of T1 latent factor

diffPCWB; !Estimate variance of latent change factor

PCWBT1; !Estimate variance of T1 latent factor

diffPCWB with PCWBT1; !Allow T1 factor to correlate with latent change factor

**Output:** Sampstat; Standardized; Cinterval;

**Model 12: Caregiver-Rated Child SDQ**

**Data:** File = RoNWBlist.dat; Type = Imputation;

**Variable:** Names are T2CGEND T1CAGEY T1CHIFB1 T1CHIFB2 T1CHIFB3 T1CHIFB4 T1CHIFB5 T1CHIFB6 T1CHIFB7 T2CHIFB1 T2CHIFB2 T2CHIFB3 T2CHIFB4 T2CHIFB5 T2CHIFB6 T2CHIFB7 T1PHIFB1 T1PHIFB2 T1PHIFB3 T1PHIFB4 T1PHIFB5 T1PHIFB6 T1PHIFB7 T2PHIFB1 T2PHIFB2 T2PHIFB3 T2PHIFB4 T2PHIFB5 T2PHIFB6 T2PHIFB7 T2THIFB1 T2THIFB2 T2THIFB3 T2THIFB4 T1PSDPRO T1PSDHYP T1PSDEMO T1PSDCON T1PSDPPR T2PSDHYP T2PSDEMO T2PSDCON T2PSDPPR T1VAZ SESZ T1EFFS T2ACADFS T2SOCFS ID;

Usevar are T1PSDHYP T1PSDEMO T1PSDCON T1PSDPPR T2PSDHYP T2PSDEMO T2PSDCON T2PSDPPR;

Missing are all (-99); IDVAR = ID;

**Analysis:** Estimator = MLR;

**Model:**

!Time 1

CMHT1 BY T1PSDHYP T1PSDEMO (1) T1PSDCON (2) T1PSDPPR (3);

!Time 2

CMHT2 BY T2PSDHYP T2PSDEMO (1) T2PSDCON (2) T2PSDPPR (3);

!Intercepts

[T1PSDHYP@0]; [T1PSDEMO](4); [T1PSDCON](5); [T1PSDPPR](6);

[T2PSDHYP@0]; [T2PSDEMO](4); [T2PSDCON](5); [T2PSDPPR](6);

!Residuals

T1PSDHYP (7); T1PSDEMO (8); T1PSDCON (9); T1PSDPPR (10);

T2PSDHYP (7); T2PSDEMO (8); T2PSDCON (9); T2PSDPPR (10);

!Correlated Residuals

T1PSDHYP with T2PSDHYP;

T1PSDEMO with T2PSDEMO;

T1PSDCON with T2PSDCON;

T1PSDPPR with T2PSDPPR;

CMHT2 on CMHT1@1; !Fixed regression of T2 on T1

diffCMH BY CMHT2@1; !Latent Change Factor for Caregiver-Rated Child SDQ

[CMHT2@0]; !Fixed latent mean of T2 to 0

CMHT2@0; !Fixed latent variance of T2 to 0

[diffCMH]; !Estimate mean of latent change factor

[CMHT1]; !Estimate mean of T1 latent factor

diffCMH; !Estimate variance of latent change factor

CMHT1; !Estimate variance of T1 latent factor

diffCMH with CMHT1; !Allow T1 factor to correlate with latent change factor

**Output:** Sampstat; Standardized; Cinterval;

**Bivariate Latent Change Score Models**

**Model 13: Child-Rated HIFAMS and Caregiver-Rated Child SDQ**

**Data:** File = RoNWBlist.dat; Type = Imputation;

**Variable:** Names are T2CGEND T1CAGEY T1CHIFB1 T1CHIFB2 T1CHIFB3 T1CHIFB4 T1CHIFB5 T1CHIFB6 T1CHIFB7 T2CHIFB1 T2CHIFB2 T2CHIFB3 T2CHIFB4 T2CHIFB5 T2CHIFB6 T2CHIFB7 T1PHIFB1 T1PHIFB2 T1PHIFB3 T1PHIFB4 T1PHIFB5 T1PHIFB6 T1PHIFB7 T2PHIFB1 T2PHIFB2 T2PHIFB3 T2PHIFB4 T2PHIFB5 T2PHIFB6 T2PHIFB7 T2THIFB1 T2THIFB2 T2THIFB3 T2THIFB4 T1PSDPRO T1PSDHYP T1PSDEMO T1PSDCON T1PSDPPR T2PSDHYP T2PSDEMO T2PSDCON T2PSDPPR T1VAZ SESZ T1EFFS T2ACADFS T2SOCFS ID;

Usevar are T1PSDHYP T1PSDEMO T1PSDCON T1PSDPPR T2PSDHYP T2PSDEMO T2PSDCON T2PSDPPR T1CHIFB1 T1CHIFB2 T1CHIFB3 T1CHIFB4 T1CHIFB5 T1CHIFB6 T1CHIFB7 T2CHIFB1T2CHIFB2 T2CHIFB3 T2CHIFB4 T2CHIFB5 T2CHIFB6 T2CHIFB7;

Categorical are T1CHIFB1 T1CHIFB2 T1CHIFB3 T1CHIFB4 T1CHIFB5 T1CHIFB6 T1CHIFB7 T2CHIFB1T2CHIFB2 T2CHIFB3 T2CHIFB4 T2CHIFB5 T2CHIFB6 T2CHIFB7;

Missing are all (-99); IDVAR = ID;

**Analysis:** Estimator = WLSMV;

**Model:**

!Caregiver-Rated Child SDQ

!Time 1

CMHT1 BY T1PSDHYP T1PSDEMO (1) T1PSDCON (2) T1PSDPPR (3);

!Time 2

CMHT2 BY T2PSDHYP T2PSDEMO (1) T2PSDCON (2) T2PSDPPR (3);

!Intercepts

[T1PSDHYP@0]; [T1PSDEMO](4); [T1PSDCON](5); [T1PSDPPR](6);

[T2PSDHYP@0]; [T2PSDEMO](4); [T2PSDCON](5); [T2PSDPPR](6);

!Residuals

T1PSDHYP (7); T1PSDEMO (8); T1PSDCON (9); T1PSDPPR (10);

T2PSDHYP (7); T2PSDEMO (8); T2PSDCON (9); T2PSDPPR (10);

!Correlated Residuals

T1PSDHYP with T2PSDHYP;

T1PSDEMO with T2PSDEMO;

T1PSDCON with T2PSDCON;

T1PSDPPR with T2PSDPPR;

CMHT2 on CMHT1@1; !Fixed regression of T2 on T1

diffCMH BY CMHT2@1; !Latent Change Factor for Caregiver-Rated Child SDQ

[CMHT2@0]; !Fixed latent mean of T2 to 0

CMHT2@0; !Fixed latent variance of T2 to 0

[diffCMH]; !Estimate mean of latent change factor

[CMHT1]; !Estimate mean of T1 latent factor

diffCMH; !Estimate variance of latent change factor

CMHT1; !Estimate variance of T1 latent factor

!Child-Rated HIFAMS

!Time 1

CWBT1 BY T1CHIFB1 T1CHIFB2 (1) T1CHIFB3 (2) T1CHIFB4 (3) T1CHIFB5 (4) T1CHIFB6 (5) T1CHIFB7 (6);

!Time 2

CWBT2 BY T2CHIFB1 T2CHIFB2 (1) T2CHIFB3 (2) T2CHIFB4 (3) T2CHIFB5 (4) T2CHIFB6 (5) T2CHIFB7 (6);

!Thresholds

[T1CHIFB1$1@0]; [T1CHIFB2$1] ; [T1CHIFB3$1] (8); [T1CHIFB4$1] (9); [T1CHIFB5$1] (10); [T1CHIFB6$1] ; [T1CHIFB7$1] (12); [T2CHIFB1$1@0]; [T2CHIFB2$1] ; [T2CHIFB3$1] (8); [T2CHIFB4$1] (9); [T2CHIFB5$1] (10); [T2CHIFB6$1] ; [T2CHIFB7$1] (12);

!Correlated Residuals

T1CHIFB1 with T2CHIFB1; T1CHIFB2 with T2CHIFB2; T1CHIFB3 with T2CHIFB3; T1CHIFB4 with T2CHIFB4; T1CHIFB5 with T2CHIFB5; T1CHIFB6 with T2CHIFB6; T1CHIFB7 with T2CHIFB7;

CWBT2 on CWBT1@1; !Fixed regression of T2 on T1

diffCWB BY CWBT2@1; !Latent Change Factor for Child Wellbeing

[CWBT2@0]; !Fixed latent mean of T2 to 0

CWBT2@0; !Fixed latent variance of T2 to 0

[diffCWB]; !Estimate mean of latent change factor

[CWBT1]; !Estimate mean of T1 latent factor

diffCWB; !Estimate variance of latent change factor

CWBT1; !Estimate variance of T1 latent factor

!Cross-Domain Associations

diffCMH with diffCWB; !Correlated latent change factors

CMHT1 with CWBT1; !Correlated between T1 latent factors

diffCMH on CWBT1!Change in mental health regressed onto initial wellbeing

diffCWB on CMHT1!Change in wellbeing regressed onto initial mental health

diffCMH on CMHT1; !Change in mental health regressed onto initial mental health

diffCWB on CWBT1; !Change in wellbeing regressed onto initial wellbeing

**Output:** Sampstat; Standardized; Cinterval;

**Model 14: Caregiver-Rated Child HIFAMS and Caregiver-Rated Child SDQ**

**Data:** File = RoNWBlist.dat; Type = Imputation;

**Variable:** Names are T2CGEND T1CAGEY T1CHIFB1 T1CHIFB2 T1CHIFB3 T1CHIFB4 T1CHIFB5 T1CHIFB6 T1CHIFB7 T2CHIFB1 T2CHIFB2 T2CHIFB3 T2CHIFB4 T2CHIFB5 T2CHIFB6 T2CHIFB7 T1PHIFB1 T1PHIFB2 T1PHIFB3 T1PHIFB4 T1PHIFB5 T1PHIFB6 T1PHIFB7 T2PHIFB1 T2PHIFB2 T2PHIFB3 T2PHIFB4 T2PHIFB5 T2PHIFB6 T2PHIFB7 T2THIFB1 T2THIFB2 T2THIFB3 T2THIFB4 T1PSDPRO T1PSDHYP T1PSDEMO T1PSDCON T1PSDPPR T2PSDHYP T2PSDEMO T2PSDCON T2PSDPPR T1VAZ SESZ T1EFFS T2ACADFS T2SOCFS ID;

Usevar are T1PSDHYP T1PSDEMO T1PSDCON T1PSDPPR T2PSDHYP T2PSDEMO T2PSDCON T2PSDPPR T1PHIFB1 T1PHIFB2 T1PHIFB3 T1PHIFB4 T1PHIFB5 T1PHIFB6 T1PHIFB7 T2PHIFB1T2PHIFB2 T2PHIFB3 T2PHIFB4 T2PHIFB5 T2PHIFB6 T2PHIFB7;

Categorical are T1PHIFB1 T1PHIFB2 T1PHIFB3 T1PHIFB4 T1PHIFB5 T1PHIFB6 T1PHIFB7 T2PHIFB1T2PHIFB2 T2PHIFB3 T2PHIFB4 T2PHIFB5 T2PHIFB6 T2PHIFB7;

Missing are all (-99); IDVAR = ID;

**Analysis:** Estimator = WLSMV;

**Model:**

!Caregiver-Rated Child SDQ

!Time 1

CMHT1 BY T1PSDHYP T1PSDEMO (1) T1PSDCON (2) T1PSDPPR (3);

!Time 2

CMHT2 BY T2PSDHYP T2PSDEMO (1) T2PSDCON (2) T2PSDPPR (3);

!Intercepts

[T1PSDHYP@0]; [T1PSDEMO](4); [T1PSDCON](5); [T1PSDPPR](6);

[T2PSDHYP@0]; [T2PSDEMO](4); [T2PSDCON](5); [T2PSDPPR](6);

!Residuals

T1PSDHYP (7); T1PSDEMO (8); T1PSDCON (9); T1PSDPPR (10);

T2PSDHYP (7); T2PSDEMO (8); T2PSDCON (9); T2PSDPPR (10);

!Correlated Residuals

T1PSDHYP with T2PSDHYP;

T1PSDEMO with T2PSDEMO;

T1PSDCON with T2PSDCON;

T1PSDPPR with T2PSDPPR;

CMHT2 on CMHT1@1; !Fixed regression of T2 on T1

diffCMH BY CMHT2@1; !Latent Change Factor for Caregiver-Rated Child SDQ

[CMHT2@0]; !Fixed latent mean of T2 to 0

CMHT2@0; !Fixed latent variance of T2 to 0

[diffCMH]; !Estimate mean of latent change factor

[CMHT1]; !Estimate mean of T1 latent factor

diffCMH; !Estimate variance of latent change factor

CMHT1; !Estimate variance of T1 latent factor

!Caregiver-Rated HIFAMS

!Time 1

PCWBT1 BY T1PHIFB1 T1PHIFB2 (a) T1PHIFB3 (b) T1PHIFB4 (c) T1PHIFB5 (d) T1PHIFB6 (e) T1PHIFB7 (f); T1PHIFB4 with T1PHIFB5 (g);

!Time 2

PCWBT2 BY T2PHIFB1T2PHIFB2 (a) T2PHIFB3 (b) T2PHIFB4 (c) T2PHIFB5 (d) T2PHIFB6 (e) T2PHIFB7 (f); T2PHIFB4 with T2PHIFB5 (g);

!Thresholds

[T1PHIFB1$1@0]; [T1PHIFB2$1] (h); [T1PHIFB3$1] (i); [T1PHIFB4$1] (j); [T1PHIFB5$1] (k); [T1PHIFB6$1] (l); [T1PHIFB7$1] (m); [T2PHIFB1$1@0]; [T2PHIFB2$1] (h); [T2PHIFB3$1] (i); [T2PHIFB4$1] (j); [T T2PHIFB5$1] (k) ; [T2PHIFB6$1] (l); [T2PHIFB7$1] (m) ;

!Correlated Residuals

T1PHIFB1 with T2PHIFB1; T1PHIFB2 with T2PHIFB2; T1PHIFB3 with T2PHIFB3; T1PHIFB4 with T2PHIFB4; T1PHIFB5 with T2PHIFB5; T1PHIFB6 with T2PHIFB6; T1PHIFB7 with T2PHIFB7;

PCWBT2 on PCWBT1@1; !Fixed regression of T2 on T1

diffPCWB BY PCWBT2@1; !Latent Change Factor for Caregiver-Rated Child Wellbeing

[PCWBT2@0]; !Fixed latent mean of T2 to 0

PCWBT2@0; !Fixed latent variance of T2 to 0

[diffPCWB]; !Estimate mean of latent change factor

[PCWBT1]; !Estimate mean of T1 latent factor

diffPCWB; !Estimate variance of latent change factor

PCWBT1; !Estimate variance of T1 latent factor

diffPCWB with PCWBT1; !Allow T1 factor to correlate with latent change factor

!Cross-Domain Associations

diffCMH with diffPCWB; !Correlated latent change factors

CMHT1 with PCWBT1; !Correlated between T1 latent factors

diffCMH on PCWBT1!Change in mental health regressed onto initial wellbeing

diffPCWB on CMHT1!Change in wellbeing regressed onto initial mental health

diffCMH on CMHT1; !Change in mental health regressed onto initial mental health

diffPCWB on PCWBT1; !Change in wellbeing regressed onto initial wellbeing

**Output:** Sampstat; Standardized; Cinterval;

**Models Predicting Social and Academic Outcomes**

**Model 15: Caregiver-Rated Mental Health 🡪 Academic Skills**

!Full information maximum likelihood estimation was used as the model provided a better fit to the data than the model using multiple imputation.

**Variable:** Names are T2CGEND T1CAGEY T1CHIFB1 T1CHIFB2 T1CHIFB3 T1CHIFB4 T1CHIFB5 T1CHIFB6 T1CHIFB7 T2CHIFB1 T2CHIFB2 T2CHIFB3 T2CHIFB4 T2CHIFB5 T2CHIFB6 T2CHIFB7 T1PHIFB1 T1PHIFB2 T1PHIFB3 T1PHIFB4 T1PHIFB5 T1PHIFB6 T1PHIFB7 T2PHIFB1 T2PHIFB2 T2PHIFB3 T2PHIFB4 T2PHIFB5 T2PHIFB6 T2PHIFB7 T2THIFB1 T2THIFB2 T2THIFB3 T2THIFB4 T1PSDPRO T1PSDHYP T1PSDEMO T1PSDCON T1PSDPPR T2PSDHYP T2PSDEMO T2PSDCON T2PSDPPR T1VAZ SESZ T1EFFS T2ACADFS T2SOCFS ID;

Usevar are T1PSDPRO T1PSDHYP T1PSDEMO T1PSDCON T1PSDPPR T2PSDHYP T2PSDEMO T2PSDCON T2PSDPPR T2CGEND T1VAZ SESZ T1EFFS T2ACADFS;

Missing are all (-99); IDVAR = ID;

**Analysis:** Estimator = MLR;

**Model:**

!Caregiver-Rated Child SDQ

!Time 1

CMHT1 BY T1PSDHYP T1PSDEMO (1) T1PSDCON (2) T1PSDPPR (3);

!Time 2

CMHT2 BY T2PSDHYP T2PSDEMO (1) T2PSDCON (2) T2PSDPPR (3);

!Intercepts

[T1PSDHYP@0]; [T1PSDEMO](4); [T1PSDCON](5); [T1PSDPPR](6);

[T2PSDHYP@0]; [T2PSDEMO](4); [T2PSDCON](5); [T2PSDPPR](6);

!Residuals

T1PSDHYP (7); T1PSDEMO (8); T1PSDCON (9); T1PSDPPR (10);

T2PSDHYP (7); T2PSDEMO (8); T2PSDCON (9); T2PSDPPR (10);

!Correlated Residuals

T1PSDHYP with T2PSDHYP;

T1PSDEMO with T2PSDEMO;

T1PSDCON with T2PSDCON;

T1PSDPPR with T2PSDPPR;

CMHT2 on CMHT1@1; !Fixed regression of T2 on T1

diffCMH BY CMHT2@1; !Latent Change Factor for Caregiver-Rated Child SDQ

[CMHT2@0]; !Fixed latent mean of T2 to 0

CMHT2@0; !Fixed latent variance of T2 to 0

[diffCMH]; !Estimate mean of latent change factor

[CMHT1]; !Estimate mean of T1 latent factor

!Regress Academic Scores onto covariates

T2ACADFS on T2CGEND T1VAZ SESZ T1EFFS T1PSDPRO;

!Regress Academic Scores onto mental health

T2ACADFS on CMHT1 diffCMH;

!Covariances

CMHT1 with diffCMH;

diffCMH with T1VAZ SESZ T1EFFS T1PSDPRO;

CMHT1 with T1VAZ SESZ T1EFFS T1PSDPRO;

diffCMH on T2CGEND; CMHT1 on T2GEND;

T1PSDEMO on T2CGEND; T2PSDEMO with T2PSDPPR; !modification indices

[T1VAZ]; [SESZ]; [T1EFFS]; [T1PSDPRO];

**Output:** Sampstat; Standardized; Cinterval;

**Model 16: Caregiver-Rated Mental Health 🡪 Social Competence**

!Full information maximum likelihood estimation was used as the model provided a better fit to the data than the model using multiple imputation.

**Variable:** Names are T2CGEND T1CAGEY T1CHIFB1 T1CHIFB2 T1CHIFB3 T1CHIFB4 T1CHIFB5 T1CHIFB6 T1CHIFB7 T2CHIFB1 T2CHIFB2 T2CHIFB3 T2CHIFB4 T2CHIFB5 T2CHIFB6 T2CHIFB7 T1PHIFB1 T1PHIFB2 T1PHIFB3 T1PHIFB4 T1PHIFB5 T1PHIFB6 T1PHIFB7 T2PHIFB1 T2PHIFB2 T2PHIFB3 T2PHIFB4 T2PHIFB5 T2PHIFB6 T2PHIFB7 T2THIFB1 T2THIFB2 T2THIFB3 T2THIFB4 T1PSDPRO T1PSDHYP T1PSDEMO T1PSDCON T1PSDPPR T2PSDHYP T2PSDEMO T2PSDCON T2PSDPPR T1VAZ SESZ T1EFFS T2ACADFS T2SOCFS ID;

Usevar are T1PSDPRO T1PSDHYP T1PSDEMO T1PSDCON T1PSDPPR T2PSDHYP T2PSDEMO T2PSDCON T2PSDPPR T2CGEND T1VAZ SESZ T1EFFS T2SOCFS;

Missing are all (-99); IDVAR = ID;

**Analysis:** Estimator = MLR;

**Model:**

!Caregiver-Rated Child SDQ

!Time 1

CMHT1 BY T1PSDHYP T1PSDEMO (1) T1PSDCON (2) T1PSDPPR (3);

!Time 2

CMHT2 BY T2PSDHYP T2PSDEMO (1) T2PSDCON (2) T2PSDPPR (3);

!Intercepts

[T1PSDHYP@0]; [T1PSDEMO](4); [T1PSDCON](5); [T1PSDPPR](6);

[T2PSDHYP@0]; [T2PSDEMO](4); [T2PSDCON](5); [T2PSDPPR](6);

!Residuals

T1PSDHYP (7); T1PSDEMO (8); T1PSDCON (9); T1PSDPPR (10);

T2PSDHYP (7); T2PSDEMO (8); T2PSDCON (9); T2PSDPPR (10);

!Correlated Residuals

T1PSDHYP with T2PSDHYP;

T1PSDEMO with T2PSDEMO;

T1PSDCON with T2PSDCON;

T1PSDPPR with T2PSDPPR;

CMHT2 on CMHT1@1; !Fixed regression of T2 on T1

diffCMH BY CMHT2@1; !Latent Change Factor for Caregiver-Rated Child SDQ

[CMHT2@0]; !Fixed latent mean of T2 to 0

CMHT2@0; !Fixed latent variance of T2 to 0

[diffCMH]; !Estimate mean of latent change factor

[CMHT1]; !Estimate mean of T1 latent factor

!Regress Social Competence Scores onto covariates

T2SOCFS on T2CGEND T1VAZ SESZ T1EFFS T1PSDPRO;

!Regress Social Competence Scores onto mental health

T2SOCFS on CMHT1 diffCMH;

!Covariances

CMHT1 with diffCMH;

diffCMH with T1VAZ SESZ T1EFFS T1PSDPRO;

CMHT1 with T1VAZ SESZ T1EFFS T1PSDPRO;

diffCMH on T2CGEND; CMHT1 on T2GEND;

T1PSDEMO on T2CGEND; T2PSDEMO with T2PSDPPR; !modification indices

[T1VAZ]; [SESZ]; [T1EFFS]; [T1PSDPRO];

**Output:** Sampstat; Standardized; Cinterval;

**Model 17: Caregiver-Rated Child Wellbeing 🡪 Academic Skills**

**Data:** File = RoNWBlist.dat; Type = Imputation;

**Variable:** Names are T2CGEND T1CAGEY T1CHIFB1 T1CHIFB2 T1CHIFB3 T1CHIFB4 T1CHIFB5 T1CHIFB6 T1CHIFB7 T2CHIFB1 T2CHIFB2 T2CHIFB3 T2CHIFB4 T2CHIFB5 T2CHIFB6 T2CHIFB7 T1PHIFB1 T1PHIFB2 T1PHIFB3 T1PHIFB4 T1PHIFB5 T1PHIFB6 T1PHIFB7 T2PHIFB1 T2PHIFB2 T2PHIFB3 T2PHIFB4 T2PHIFB5 T2PHIFB6 T2PHIFB7 T2THIFB1 T2THIFB2 T2THIFB3 T2THIFB4 T1PSDPRO T1PSDHYP T1PSDEMO T1PSDCON T1PSDPPR T2PSDHYP T2PSDEMO T2PSDCON T2PSDPPR T1VAZ SESZ T1EFFS T2ACADFS T2SOCFS ID;

Usevar are T1PSDPRO T1PHIFB1 T1PHIFB2 T1PHIFB3 T1PHIFB4 T1PHIFB5 T1PHIFB6 T1PHIFB7 T2PHIFB1T2PHIFB2 T2PHIFB3 T2PHIFB4 T2PHIFB5 T2PHIFB6 T2PHIFB7 T2CGEND T1VAZ SESZ T1EFFS T2SOCFS;

Categorical are T1PHIFB1 T1PHIFB2 T1PHIFB3 T1PHIFB4 T1PHIFB5 T1PHIFB6 T1PHIFB7 T2PHIFB1T2PHIFB2 T2PHIFB3 T2PHIFB4 T2PHIFB5 T2PHIFB6 T2PHIFB7;

Missing are all (-99); IDVAR = ID;

**Analysis:** Estimator = WLSMV;

**Model:**

!Caregiver-Rated HIFAMS

!Time 1

PCWBT1 BY T1PHIFB1 T1PHIFB2 (a) T1PHIFB3 (b) T1PHIFB4 (c) T1PHIFB5 (d) T1PHIFB6 (e) T1PHIFB7 (f); T1PHIFB4 with T1PHIFB5 (g);

!Time 2

PCWBT2 BY T2PHIFB1T2PHIFB2 (a) T2PHIFB3 (b) T2PHIFB4 (c) T2PHIFB5 (d) T2PHIFB6 (e) T2PHIFB7 (f); T2PHIFB4 with T2PHIFB5 (g);

!Thresholds

[T1PHIFB1$1@0]; [T1PHIFB2$1] (h); [T1PHIFB3$1] (i); [T1PHIFB4$1] (j); [T1PHIFB5$1] (k); [T1PHIFB6$1] (l); [T1PHIFB7$1] (m); [T2PHIFB1$1@0]; [T2PHIFB2$1] (h); [T2PHIFB3$1] (i); [T2PHIFB4$1] (j); [T T2PHIFB5$1] (k) ; [T2PHIFB6$1] (l); [T2PHIFB7$1] (m) ;

!Correlated Residuals

T1PHIFB1 with T2PHIFB1; T1PHIFB2 with T2PHIFB2; T1PHIFB3 with T2PHIFB3; T1PHIFB4 with T2PHIFB4; T1PHIFB5 with T2PHIFB5; T1PHIFB6 with T2PHIFB6; T1PHIFB7 with T2PHIFB7;

PCWBT2 on PCWBT1@1; !Fixed regression of T2 on T1

diffPCWB BY PCWBT2@1; !Latent Change Factor for Caregiver-Rated Child Wellbeing

[PCWBT2@0]; !Fixed latent mean of T2 to 0

PCWBT2@0; !Fixed latent variance of T2 to 0

[diffPCWB]; !Estimate mean of latent change factor

[PCWBT1]; !Estimate mean of T1 latent factor

!Regress Academic Scores onto covariates

T2ACADFS on T2CGEND T1VAZ SESZ T1EFFS T1PSDPRO;

!Regress Academic Scores onto mental health

T2ACADFS on PCWBT1 diffPCWB;

!Covariances

PCWBT1 with diffPCWB;

diffPCWB with T1VAZ SESZ T1EFFS T1PSDPRO;

PCWBT1 with T1VAZ SESZ T1EFFS T1PSDPRO;

diffPCWB on T2CGEND; PCWBT1 on T2GEND;

[T1VAZ]; [SESZ]; [T1EFFS]; [T1PSDPRO];

**Output:** Sampstat; Standardized; Cinterval;

**Model 18: Caregiver-Rated Child Wellbeing 🡪 Social Competence**

**Data:** File = RoNWBlist.dat; Type = Imputation;

**Variable:** Names are T2CGEND T1CAGEY T1CHIFB1 T1CHIFB2 T1CHIFB3 T1CHIFB4 T1CHIFB5 T1CHIFB6 T1CHIFB7 T2CHIFB1 T2CHIFB2 T2CHIFB3 T2CHIFB4 T2CHIFB5 T2CHIFB6 T2CHIFB7 T1PHIFB1 T1PHIFB2 T1PHIFB3 T1PHIFB4 T1PHIFB5 T1PHIFB6 T1PHIFB7 T2PHIFB1 T2PHIFB2 T2PHIFB3 T2PHIFB4 T2PHIFB5 T2PHIFB6 T2PHIFB7 T2THIFB1 T2THIFB2 T2THIFB3 T2THIFB4 T1PSDPRO T1PSDHYP T1PSDEMO T1PSDCON T1PSDPPR T2PSDHYP T2PSDEMO T2PSDCON T2PSDPPR T1VAZ SESZ T1EFFS T2ACADFS T2SOCFS ID;

Usevar are T1PSDPRO T1PHIFB1 T1PHIFB2 T1PHIFB3 T1PHIFB4 T1PHIFB5 T1PHIFB6 T1PHIFB7 T2PHIFB1T2PHIFB2 T2PHIFB3 T2PHIFB4 T2PHIFB5 T2PHIFB6 T2PHIFB7 T2CGEND T1VAZ SESZ T1EFFS T2SOCFS;

Categorical are T1PHIFB1 T1PHIFB2 T1PHIFB3 T1PHIFB4 T1PHIFB5 T1PHIFB6 T1PHIFB7 T2PHIFB1T2PHIFB2 T2PHIFB3 T2PHIFB4 T2PHIFB5 T2PHIFB6 T2PHIFB7;

Missing are all (-99); IDVAR = ID;

**Analysis:** Estimator = WLSMV;

**Model:**

!Caregiver-Rated HIFAMS

!Time 1

PCWBT1 BY T1PHIFB1 T1PHIFB2 (a) T1PHIFB3 (b) T1PHIFB4 (c) T1PHIFB5 (d) T1PHIFB6 (e) T1PHIFB7 (f); T1PHIFB4 with T1PHIFB5 (g);

!Time 2

PCWBT2 BY T2PHIFB1T2PHIFB2 (a) T2PHIFB3 (b) T2PHIFB4 (c) T2PHIFB5 (d) T2PHIFB6 (e) T2PHIFB7 (f); T2PHIFB4 with T2PHIFB5 (g);

!Thresholds

[T1PHIFB1$1@0]; [T1PHIFB2$1] (h); [T1PHIFB3$1] (i); [T1PHIFB4$1] (j); [T1PHIFB5$1] (k); [T1PHIFB6$1] (l); [T1PHIFB7$1] (m); [T2PHIFB1$1@0]; [T2PHIFB2$1] (h); [T2PHIFB3$1] (i); [T2PHIFB4$1] (j); [T T2PHIFB5$1] (k) ; [T2PHIFB6$1] (l); [T2PHIFB7$1] (m) ;

!Correlated Residuals

T1PHIFB1 with T2PHIFB1; T1PHIFB2 with T2PHIFB2; T1PHIFB3 with T2PHIFB3; T1PHIFB4 with T2PHIFB4; T1PHIFB5 with T2PHIFB5; T1PHIFB6 with T2PHIFB6; T1PHIFB7 with T2PHIFB7;

PCWBT2 on PCWBT1@1; !Fixed regression of T2 on T1

diffPCWB BY PCWBT2@1; !Latent Change Factor for Caregiver-Rated Child Wellbeing

[PCWBT2@0]; !Fixed latent mean of T2 to 0

PCWBT2@0; !Fixed latent variance of T2 to 0

[diffPCWB]; !Estimate mean of latent change factor

[PCWBT1]; !Estimate mean of T1 latent factor

!Regress Social Competence Scores onto covariates

T2SOCFS on T2CGEND T1VAZ SESZ T1EFFS T1PSDPRO;

!Regress Social Competence Scores onto mental health

T2SOCFS on PCWBT1 diffPCWB;

!Covariances

PCWBT1 with diffPCWB;

diffPCWB with T1VAZ SESZ T1EFFS T1PSDPRO;

PCWBT1 with T1VAZ SESZ T1EFFS T1PSDPRO;

diffPCWB on T2CGEND; PCWBT1 on T2GEND;

[T1VAZ]; [SESZ]; [T1EFFS]; [T1PSDPRO];

**Output:** Sampstat; Standardized; Cinterval;

**Model 19: Self-Rated Child Wellbeing 🡪 Academic Skills**

**Data:** File = RoNWBlist.dat; Type = Imputation;

**Variable:** Names are T2CGEND T1CAGEY T1CHIFB1 T1CHIFB2 T1CHIFB3 T1CHIFB4 T1CHIFB5 T1CHIFB6 T1CHIFB7 T2CHIFB1 T2CHIFB2 T2CHIFB3 T2CHIFB4 T2CHIFB5 T2CHIFB6 T2CHIFB7 T1PHIFB1 T1PHIFB2 T1PHIFB3 T1PHIFB4 T1PHIFB5 T1PHIFB6 T1PHIFB7 T2PHIFB1 T2PHIFB2 T2PHIFB3 T2PHIFB4 T2PHIFB5 T2PHIFB6 T2PHIFB7 T2THIFB1 T2THIFB2 T2THIFB3 T2THIFB4 T1PSDPRO T1PSDHYP T1PSDEMO T1PSDCON T1PSDPPR T2PSDHYP T2PSDEMO T2PSDCON T2PSDPPR T1VAZ SESZ T1EFFS T2ACADFS T2SOCFS ID;

Usevar are T1PSDPRO T1CHIFB1 T1CHIFB2 T1CHIFB3 T1CHIFB4 T1CHIFB5 T1CHIFB6 T1CHIFB7 T2CHIFB1 T2CHIFB2 T2CHIFB3 T2CHIFB4 T2CHIFB5 T2CHIFB6 T2CHIFB7 T2CGEND T1VAZ SESZ T1EFFS T2ACADFS;

Categorical are T1CHIFB1 T1CHIFB2 T1CHIFB3 T1CHIFB4 T1CHIFB5 T1CHIFB6 T1CHIFB7 T2CHIFB1 T2CHIFB2 T2CHIFB3 T2CHIFB4 T2CHIFB5 T2CHIFB6 T2CHIFB7;

Missing are all (-99); IDVAR = ID;

**Analysis:** Estimator = WLSMV;

**Model:**

!Time 1

CWBT1 BY T1CHIFB1 T1CHIFB2 (1) T1CHIFB3 (2) T1CHIFB4 (3) T1CHIFB5 (4) T1CHIFB6 (5) T1CHIFB7 (6);

!Time 2

CWBT2 BY T2CHIFB1 T2CHIFB2 (1) T2CHIFB3 (2) T2CHIFB4 (3) T2CHIFB5 (4) T2CHIFB6 (5) T2CHIFB7 (6);

!Thresholds

[T1CHIFB1$1@0]; [T1CHIFB2$1] ; [T1CHIFB3$1] (8); [T1CHIFB4$1] (9); [T1CHIFB5$1] (10); [T1CHIFB6$1] ; [T1CHIFB7$1] (12); [T2CHIFB1$1@0]; [T2CHIFB2$1] ; [T2CHIFB3$1] (8); [T2CHIFB4$1] (9); [T2CHIFB5$1] (10); [T2CHIFB6$1] ; [T2CHIFB7$1] (12);

!Correlated Residuals

T1CHIFB1 with T2CHIFB1; T1CHIFB2 with T2CHIFB2; T1CHIFB3 with T2CHIFB3; T1CHIFB4 with T2CHIFB4; T1CHIFB5 with T2CHIFB5; T1CHIFB6 with T2CHIFB6; T1CHIFB7 with T2CHIFB7;

CWBT2 on CWBT1@1; !Fixed regression of T2 on T1

diffCWB BY CWBT2@1; !Latent Change Factor for Child Wellbeing

[CWBT2@0]; !Fixed latent mean of T2 to 0

CWBT2@0; !Fixed latent variance of T2 to 0

[diffCWB]; !Estimate mean of latent change factor

[CWBT1]; !Estimate mean of T1 latent factor

!Regress Academic Scores onto covariates

T2ACADFS on T2CGEND T1VAZ SESZ T1EFFS T1PSDPRO;

!Regress Academic Scores onto mental health

T2ACADFS on CWBT1 diffCWB;

!Covariances

CWBT1 with diffCWB;

diffCWB with T1VAZ SESZ T1EFFS T1PSDPRO;

CWBT1 with T1VAZ SESZ T1EFFS T1PSDPRO;

diffCWB on T2CGEND; CWBT1 on T2GEND;

[T1VAZ]; [SESZ]; [T1EFFS]; [T1PSDPRO];

**Output:** Sampstat; Standardized; Cinterval;

**Model 20: Self-Rated Child Wellbeing 🡪 Social Competence**

**Data:** File = RoNWBlist.dat; Type = Imputation;

**Variable:** Names are T2CGEND T1CAGEY T1CHIFB1 T1CHIFB2 T1CHIFB3 T1CHIFB4 T1CHIFB5 T1CHIFB6 T1CHIFB7 T2CHIFB1 T2CHIFB2 T2CHIFB3 T2CHIFB4 T2CHIFB5 T2CHIFB6 T2CHIFB7 T1PHIFB1 T1PHIFB2 T1PHIFB3 T1PHIFB4 T1PHIFB5 T1PHIFB6 T1PHIFB7 T2PHIFB1 T2PHIFB2 T2PHIFB3 T2PHIFB4 T2PHIFB5 T2PHIFB6 T2PHIFB7 T2THIFB1 T2THIFB2 T2THIFB3 T2THIFB4 T1PSDPRO T1PSDHYP T1PSDEMO T1PSDCON T1PSDPPR T2PSDHYP T2PSDEMO T2PSDCON T2PSDPPR T1VAZ SESZ T1EFFS T2ACADFS T2SOCFS ID;

Usevar are T1PSDPRO T1CHIFB1 T1CHIFB2 T1CHIFB3 T1CHIFB4 T1CHIFB5 T1CHIFB6 T1CHIFB7 T2CHIFB1 T2CHIFB2 T2CHIFB3 T2CHIFB4 T2CHIFB5 T2CHIFB6 T2CHIFB7 T2CGEND T1VAZ SESZ T1EFFS T2SOCFS;

Categorical are T1CHIFB1 T1CHIFB2 T1CHIFB3 T1CHIFB4 T1CHIFB5 T1CHIFB6 T1CHIFB7 T2CHIFB1 T2CHIFB2 T2CHIFB3 T2CHIFB4 T2CHIFB5 T2CHIFB6 T2CHIFB7;

Missing are all (-99); IDVAR = ID;

**Analysis:** Estimator = WLSMV;

**Model:**

!Time 1

CWBT1 BY T1CHIFB1 T1CHIFB2 (1) T1CHIFB3 (2) T1CHIFB4 (3) T1CHIFB5 (4) T1CHIFB6 (5) T1CHIFB7 (6);

!Time 2

CWBT2 BY T2CHIFB1 T2CHIFB2 (1) T2CHIFB3 (2) T2CHIFB4 (3) T2CHIFB5 (4) T2CHIFB6 (5) T2CHIFB7 (6);

!Thresholds

[T1CHIFB1$1@0]; [T1CHIFB2$1] ; [T1CHIFB3$1] (8); [T1CHIFB4$1] (9); [T1CHIFB5$1] (10); [T1CHIFB6$1] ; [T1CHIFB7$1] (12); [T2CHIFB1$1@0]; [T2CHIFB2$1] ; [T2CHIFB3$1] (8); [T2CHIFB4$1] (9); [T2CHIFB5$1] (10); [T2CHIFB6$1] ; [T2CHIFB7$1] (12);

!Correlated Residuals

T1CHIFB1 with T2CHIFB1; T1CHIFB2 with T2CHIFB2; T1CHIFB3 with T2CHIFB3; T1CHIFB4 with T2CHIFB4; T1CHIFB5 with T2CHIFB5; T1CHIFB6 with T2CHIFB6; T1CHIFB7 with T2CHIFB7;

CWBT2 on CWBT1@1; !Fixed regression of T2 on T1

diffCWB BY CWBT2@1; !Latent Change Factor for Child Wellbeing

[CWBT2@0]; !Fixed latent mean of T2 to 0

CWBT2@0; !Fixed latent variance of T2 to 0

[diffCWB]; !Estimate mean of latent change factor

[CWBT1]; !Estimate mean of T1 latent factor

!Regress Social Competence Scores onto covariates

T2SOCFS on T2CGEND T1VAZ SESZ T1EFFS T1PSDPRO;

!Regress Social Competence Scores onto mental health

T2SOCFS on CWBT1 diffCWB;

!Covariances

CWBT1 with diffCWB;

diffCWB with T1VAZ SESZ T1EFFS T1PSDPRO;

CWBT1 with T1VAZ SESZ T1EFFS T1PSDPRO;

diffCWB on T2CGEND; CWBT1 on T2GEND;

[T1VAZ]; [SESZ]; [T1EFFS]; [T1PSDPRO];

**Output:** Sampstat; Standardized; Cinterval;
